# Effects of Ready-to-Eat-Cereals on Key Nutritional and Health Outcomes: A Systematic Review

**DOI:** 10.1371/journal.pone.0164931

**Published:** 2016-10-17

**Authors:** Marion G. Priebe, Jolene R. McMonagle

**Affiliations:** 1 University Medical Center Groningen, University of Groningen, Center for Medical Biomics, Groningen, The Netherlands; 2 Nutrition Reviewed, Murnau, Germany; 3 Cereal Partners Worldwide, Lausanne 1008, Switzerland; TNO, NETHERLANDS

## Abstract

**Background:**

In many countries breakfast cereals are an important component of breakfast. This systematic review assesses the contribution of consumption of ready-to eat cereal (RTEC) to the recommended nutrient intake. Furthermore, the effects of RTEC consumption on key health parameters are investigated as well as health promoting properties of RTEC.

**Method:**

The Cochrane Central Register of Controlled Trials, MEDLINE, EMBASE and CINAHL have been searched up till 16^th^ of June 2015. Randomized controlled trials were excluded if RTEC were used during hypocaloric diets, if RTEC were eaten at other times than breakfast and if breakfasts included other products than RTEC, milk and fruit. Observational studies were excluded when “breakfast cereals” were not defined or their definition included cooked cereals. From cross-sectional studies only data concerning energy and nutrient intake as well as micronutrient status were used.

**Results:**

From 4727 identified citations 64 publications met the inclusion criteria of which 32 were cross-sectional studies, eight prospective studies and 24 randomized controlled trials. Consumption of RTEC is associated with a healthier dietary pattern, concerning intake of carbohydrates, dietary fiber, fat and micronutrients, however total sugar intake is higher. Persons consuming RTEC frequently (≥ 5 times/week) have a lower risk of inadequate micronutrient intake especially for vitamin A, calcium, folate, vitamin B 6, magnesium and zinc. Evidence from prospective studies suggests that whole grain RTEC may have beneficial effects on hypertension and type 2 diabetes. Consumption of RTEC with soluble fiber helps to reduce LDL cholesterol in hypercholesterolemic men and RTEC fortified with folate can reduce plasma homocysteine.

**Discussion:**

One of the review’s strengths is its thorough ex/inclusion of studies. Limitations are that results of observational studies were based on self-reported data and that many studies were funded by food-industry.

**Conclusion:**

Consumption of RTEC, especially of fiber-rich or whole grain RTEC, is implicated with several beneficial nutritional and health outcomes. The effect on body weight, intestinal health and cognitive function needs further evaluation. Of concern is the higher total sugar intake associated with frequent RTEC consumption.

## Introduction

Extensive research has been shown that eating breakfast compared to skipping breakfast results in improved macro- and micro-nutrient intake and status [1], can reduce the risk of weight gain [2] and has beneficial effects on cognitive and academic performance [1;3] and development of diseases such as type 2 diabetes [4] and cardiovascular diseases [5;6]. In many countries breakfast cereals (BC) are considered the main component of a balanced breakfast. A considerable number of studies are conducted to investigate the impact of the consumption of BC on nutritional and health benefits [7–12]. In addition, several reviews summarize their effects on either specific health outcomes [13;14] or comprehensively on nutritional and health benefits [15]. The group of BC comprises many different cereal products and can be divided roughly into cooked cereals, like porridge type breakfasts, and ready-to-eat cereals (RTEC) or “cold” breakfast cereals like corn flakes and muesli. It is obvious that nutritional and health benefits depend on the composition of the breakfast meal. Many observational studies do not differentiate between RTEC and cooked cereals and in intervention trials BC are often either only part of breakfast or consumed not only for breakfast. To obtain more specifically information on nutritional and health benefits of cereals consumed at breakfast it is necessary to consider the specific composition of BC while summarizing and evaluating the available evidence. Therefore, in this systematic review, studies are included that investigate the effect of RTEC only and an attempt is made to relate their specific composition to specific health benefits.

Two questions are addressed:

1. To what extent does consumption of RTEC contribute to the recommended nutrient intake of children, adolescents and adults?
2. What are the effects of RTEC consumption on key health parameters in healthy persons as well as in persons at risk of disease and what are health promoting properties of RTEC?

Data from all available observational cohort studies and (randomized) controlled trials (RCTs) have been systematically reviewed and summarized. “Key health parameters” assessed were outcomes related to energy metabolism, weight management, cardiovascular health, digestion/gut health, immune function, performance, bone growth and development. RCTs compared either the health effect of consuming different amounts of RTEC or different types of RTEC (e.g. high- vs low-fiber RTEC). Data from cross-sectional studies have not been considered for assessing the effect on health parameters due to their limited strength of evidence.

## Methods

A protocol of this systematic review is available as supporting information (S1 Protocol). The Preferred Reporting Items for Systematic reviews and Meta-Analyses (PRISMA) and the guidelines for the Meta-analysis of Observational Studies in Epidemiology (MOOSE) were followed [16;17]. The PRISMA and MOOSE checklists are available as supporting information (S1 and S2 Checklists). Due to the low number and the diversity of the studies addressing one specific health outcome a meta-analysis was not carried out.

### Data sources and literature search

The Cochrane Central Register of Controlled Trials, MEDLINE, EMBASE and CINAHL have been searched with no time limit and no language restriction on 26^th^ of February 2014 by MGP (investigator). The following MEDLINE search strategy has been used and adapted for the other electronic databases searched: 1) Breakfast OR fortified OR ready-to-eat, 2) Cereal OR cereals, 3) 1 AND 2. No specific search items have been used for nutritional and health outcomes as we aimed for a wide range. The search was updated with the identical search-strategy on 16^th^ of June 2015. In addition, the reference lists of all included studies and of review articles have been searched in order to identify additional studies of interest. The title and abstract of each record of the search have been assessed by two reviewers (MGP, JRM) independently. Studies have been rejected if the article, based on the abstract, definitely did not meet the review's inclusion criteria, otherwise the full text of the study has been obtained and screened. Abstracts for which no full text papers were available were excluded. Differences between reviewers' results have been resolved by discussion. Studies were included if they were RCTs or prospective studies and if they assessed energy and nutrient intake or outcomes related to energy metabolism, weight management, cardiovascular health, digestion/gut health, immune function, performance, bone growth and development. Cross-sectional studies were included if they assessed energy intake, nutrient intake and micronutrient status. RTEC were defined as “a cereal food that is processed to the point where it can be eaten without further preparation, as in boxed cereals”, thus cold cereals were defined to be RTEC. RCTs were excluded if they assessed breakfast skippers vs breakfast eaters, if RTEC were used as meal replacer or during hypocaloric diets, if RTEC were eaten at other times than breakfast, if breakfasts included other products than RTEC, milk and fruit and if breakfasts differed in carbohydrate content in studies comparing postprandial blood glucose and/or insulin. Observational studies were excluded when “breakfast cereals” were not defined or the definition of “breakfast cereals” included cooked cereals. From cross-sectional studies only data concerning energy and nutrient intake as well as micronutrient status were used.

### Data extraction process and assessment of risk of bias

From original reports of the studies data were extracted by one reviewer (MGP) according to pre-designed extraction forms which were validated and used already in a similar systematic review [18]. From RCTs the following data were extracted:

1. General information: title, authors, country, year of publication, funding, duplicate publication;
2. Trial characteristics: design, duration, randomizations, concealment of allocation, blinding, checking of blinding;
3. Intervention: length of intervention, dietary advice/diet provided, comparison interventions;
4. Participants: population, exclusion criteria, number (total, per compared groups), age, gender, health condition; diagnostic criteria used to define health condition, similarity groups at baseline, assessment of compliance, withdrawals/losses to follow-up;
5. Outcomes: outcomes specified above (primary and secondary outcomes of the studies);
6. Results: for outcomes and times of assessment (including a measure of variation), intention-to-treat analyses.

The following data were extracted from cohort studies:

1. General information: title, authors, country, year of publication, duplicate publication;
2. Study characteristics: design, dates of enrolment, follow-up;
3. Exposure: type, type of measurement, validation of measurement, time-points measurements;
4. Outcome: type, criteria used, type of measurement, validation of measurement;
5. Participants: number, characteristics;
6. Results: total number of cases, cases in group with lowest and highest intake, results of outcome, confounders adjusted for.

Risk of bias of RCTs and the methodological quality of prospective and cross-sectional studies were evaluated by one reviewer (MGP). The Cochrane Collaboration’s tool for assessing risk of bias [19] was used for the appraisal of RCTs. The following seven items were assessed and rated as “low”, “high” or “unclear risk” of bias: random sequence generation, allocation concealment, blinding participants and personnel, blinding of outcome assessment, incomplete outcome data, selective reporting and other bias. As “other bias” the appropriateness and methodology (washout period, analysis) of the cross-over design in cross-over studies was examined. For the assessment of the quality of observational studies the following criteria were examined [18]: methods for selecting study participants, number of appropriate confounders investigated and adjusted for; quality of method used to assess dietary intake, e.g. food frequency questionnaire with/without validation, quality of method used to assess outcome measures: e.g. self-report with/without validation, or direct measurement/medical records and additional for prospective studies: duration/completeness of follow-up.

## Results

### Description of studies

**Fig 1:**
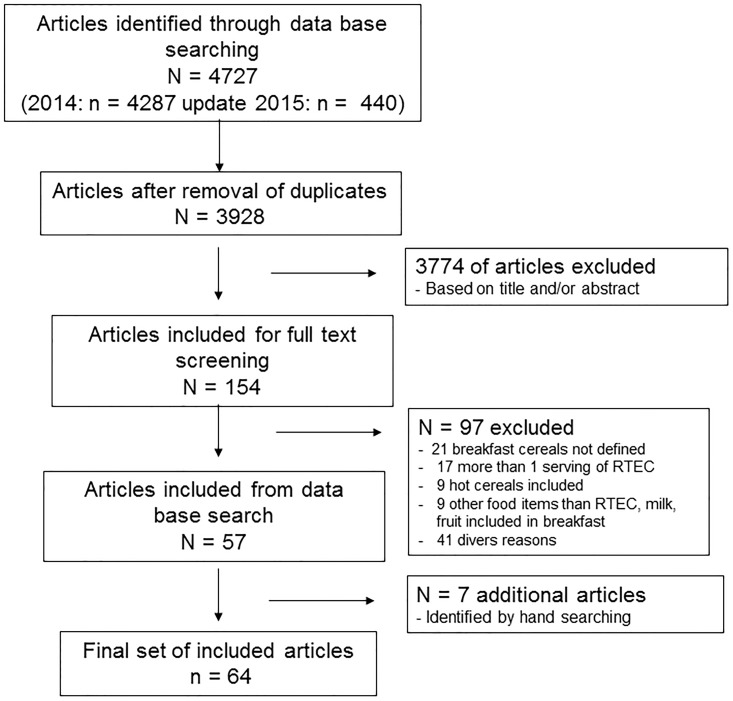
Flow diagram of articles identified in search and included in review.

The results of the literature search and the progress through the different stages of the review process are depicted in the PRISMA flow diagram (Fig 1). A total of 64 publications (all published in English) met the inclusion criteria, of which 32 were cross-sectional studies [7;9;20–49], eight prospective studies [8;50–56] and 24 RCTs [10–12;57–77].

**Fig 2:**
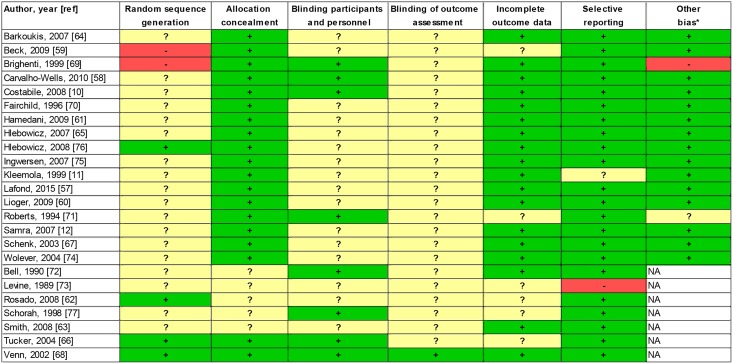
Overview risk of bias RCTs (according to Cochrane Collaboration Risk of Bias Tool). Green (+) indicates low risk of bias; Red (-) indicates high risk of bias; and Yellow (?) indicates unclear risk of bias. NA: not applicable, * for cross-over studies only.

Characteristics of cross-sectional studies are presented in Table 1, those of prospective studies in Table 2 and those of RCTs in Table 3. The impact of RTEC consumption on nutrient intake was addressed in all cross-sectional studies as well as in three prospective studies (cross-sectional at baseline [51;53] and prospectively [51]) and one RCT [11]. All RCTs and prospective studies assessed health parameters, which were risk factors for cardiovascular disease [10;11;52;54;56;58;62;66;68;69;71;72;77] and type 2 diabetes [50;55;59;60;64;67;70;74], BMI/body weight/satiety/food intake [8;11;12;51;54;57;59–62;65;73;76], digestion/gut health [10;58;69] and cognitive performance [53;63;75]. No publications were found assessing immune function and bone growth and development. The risk of bias for all individual RTCs is depicted in Fig 2. Overall the RCTs had a low or unclear risk of bias. Based on the items assessed, all selected observational studies have been judged to be of appropriate methodological quality. As many studies (45 studies, Tables 1–3) were funded by food industry, funding is also reported together with the results.

**Table 1:** Characteristics cross-sectional studies assessing associations between frequency of RTEC consumption and energy and nutrient intake.

Reference, country, sponsor	Study name, year	Cohort, age range	Method dietary assessment	Nutritional intake, categorization	Outcomes^a^
Affenito et al, 2013 [23], USA, General Mills	Third School Nutrition Dietary Assessment Study	N = 2298, 5–18 y	24-h dietary recall	SBP nonparticipants: RTEC vs no RTEC at breakfast SBP participants: RTEC vs no RTEC at breakfast	Energy, nutrient intake, percentage receiving less than EAR
Albertson and Tobelmann, 1993 [7], USA, General Mills	Market Research Corporation of America Menu Census Panel Survey 1986–1987 and 1987–1988	N = 824, 7–12 y	14-day food diary	> 7 times vs 2–6 times vs < 2 times/14 d	Energy, nutrient intake, percentage of population consuming less than 100% RDA
Albertson et al, 2003 [36], USA, General Mills		N = 603, 4–12 y	14-day food diary, minimum of 7 days had to be filled in	≥ 8 serving vs ≤3 serving/14 d	Energy, nutrient intake, percentage receiving less than EAR
Albertson et al, 2011 [27], USA, General Mills	NHANES 2001–2006	N = 9660, 6–18 y	24-h dietary recall	≥ 1 serving/day vs non	Energy, nutrient intake, percentage receiving less than EAR
Albertson et al, 2012 [25], USA, General Mills	National Eating Trends 2006–2008	N = 1759, ≥ 55 y	14-day food diary	> 8 serving vs 0 serving/14 d; to be assigned to the “whole grain” category the first grain ingredient in the product must be a whole grain.	Energy, nutrient intake, percentage receiving less than EAR
Albertson et al, 2013 [44], USA, General Mills	NHANES, 2003–2008	N = 4737, 4–12 y Food secure and not food secure	24-h dietary recall	RTEC vs no RTEC	Energy, nutrient intake, percentage receiving less than EAR
Albertson et al, 2013 [46], Canada, General Mills	2003–2004	N = 2026, ≥ 12 y	7-day food diary	≥ 4 serving vs 2–3 serving vs 0–1 serving/7 d	Energy, nutrient intake, percentage receiving less than EAR
Barr et al, 2013 [24], Canada, Kellogg	Canadian Community Health Survey 2004	n = 19913, ≥ 19 y	24-h dietary recall	RTEC vs no RTEC at breakfast	Energy, nutrient intake, percentage receiving less than EAR
Barr et al, 2014 [22], Canada, Kellogg	Canadian Community Health Survey, 2004	N = 12281, 4–18 y	24-h dietary recall	RTEC vs no RETC at breakfast	Energy, nutrient intake, percentage receiving less than EAR
Bertrais et al, 2000 [9], France, in part by Kellogg	”Supplementation en Vitamines et Minéraux Antioxydants” cohort	N = 2188 men, 45–60 y; N = 2,851 women, 35–60 y	12 x 24-h dietary recalls averaged	RTEC breakfast on 0–1 d vs 2–5 d vs 6–9 d vs 10–12 d/12 d	Energy, nutrient intake
Deshmukh-Taskar et al, 2010 [29], USA, in part by Kellogg	NHANES 1999–2006	N = 4320, 9–13 y; N = 5,339, 14–18 y	24-h dietary recall	RTEC vs no RTEC at breakfast	Energy, nutrient intake
Deshmukh-Taskar et al, 2010 [28], USA, USDA Agricultural Research Service	NHANES 1999–2002	N = 2615, 20–39 y	24-h dietary recall	RTEC vs no RTEC at breakfast	Energy, nutrient intake
Galvin et al, 2002 [37], Ireland, in part by Kellogg	North/South Ireland Food Consumption Survey 1997–1999	N = 1379, 18–64 y	7-day food diary	0 g vs on average 28 g/d	Energy, nutrient intake, percentage receiving less than EAR
Grieger et al, 2012 [26], Australia, Kellogg	Australian National Children’s Nutrition and Physical Activity Survey 2007	N = 781 boys, 12–16 y	Two 24-h food recalls	RTEC vs no RTEC at breakfast	Energy, nutrient intake, probability of not achieving 100% of EAR
Grieger et al, 2013 [45], Australia, Kellogg	Australian National Children’s Nutrition and Physical Activity Survey.2007	N = 4487, 2–16 y	Two 24-h food recalls	RTEC vs no RTEC at breakfast	Dietary fiber intake
Kafatos et al, 2005 [35], Crete, Kellogg	1992	N = 392, 15 ± 0.4 y	24-h dietary recall, FFQ for RTEC consumption	6–5 times vs 1–4 times vs < 1/week	Energy, nutrient intake
Koo et al, 2014 [21], Malaysia, Universiti Kebangsaan Malaysia		N = 382, 10–11 y	24-h dietary recall	RTEC vs no RTEC at breakfast	Energy, nutrient intake
McNulty et al, 1996 [39], Northern Ireland, Kellogg	1990/1991	N = 1015, 12 and 15 y	Dietary history method	> 40 g vs 0 g/day	Energy, nutrient intake, percentage not achieving LRNI
Montenegro-Bethancourt et al, 2009 [30], Guatemala, Kellogg	2005	N = 449 boys, 8–10 y	24-h dietary recall	RTEC vs no RTEC	Energy, nutrient intake
Morgan et al, 1981 [43], USA, not stated	1977	N = 657, 5–12 y	7-day food diaries	≥ 3 times vs < 3 times vs 0 times/7 day at breakfast	Energy, nutrient intake
Morgan et al, 1986 [48], USA, not stated	Nationwide Food Consumption Survey 1977–78	N = 11082, 1–17 y	3-day dietary record	≥ 2 times RTEC vs no RTEC/3 days at breakfast	Energy, nutrient intake
Nicklas et al, 1995 [42], USA, in part by Kellogg	Bogalusa Heart Study 1984–1985, 1987–1988 and 1988–1991	N = 568, 10 y; N = 504, 19–28 y	24-h dietary recall	RTEC vs no RTEC	Energy, nutrient intake, percentage of population receiving less than 2/3 of RDA
Ortega et al, 1996 [41], Spain, The National Institute of Diabetes and Digestive and Kidney Diseases/National Institutes of Health		N = 200, 9–13 y	weighed food record over 5 consecutive days	Every day RTEC vs never RTEC at breakfast	Energy, nutrient intake, micronutrient status
Papoutsou et al, 2014 [20], Cyprus, European Community within the Sixth RTD Framework Programme	IDEFICS study 2007–2008	N = 1558, 4–8 y	24-h dietary recall	RTEC vs no RTEC at breakfast	Energy, nutrient intake, percentage of population receiving less than 2/3 of RDA
Preziosi et al, 1999 [38], France, Kellogg		N = 1108, 2–65 y	Dietary history method	RTEC vs no RTEC at breakfast	Energy, nutrient intake, micronutrient status
Ruxton et al, 1996 [40], Scotland, Kellogg	1991	N = 136, 7–8 y	7-d weighed food record	6–7 times RTEC vs 4–5 times RTEC vs 0–3 times RTEC/week at breakfast	Energy, nutrient intake,
Sampson et al, 1995 [47], USA, General Mills	1989	N = 1151, 7–10 y	24-h dietary recall	RTEC vs no RTEC at breakfast	Energy, nutrient intake, percentage of population consuming less than 80% RDA
Song et al, 2005 [34], USA, Kellogg	NHANES 1999–2000	N = 4219, ≥ 19 y	24-h dietary recall	RTEC vs no RTEC at breakfast	Energy, nutrient intake
Song et al, 2006 [32], USA, in part by Kellogg	NHANES 1999–2000	N = 7403, ≥ 4 y	24-h dietary recall	RTEC vs no RTEC at breakfast	Energy, nutrient intake, calcium intake
Van den Boom et al, 2006 [33], Spain, Kellogg	enKid study	N = 2852 (without underreporters), 2–24 y	24-h dietary recall	> 40 g vs 21–40 g vs 1–20 g vs 0 g/day	Energy, nutrient intake, percentage of population receiving less than 2/3 of RDA
Williams et al, 2009 [31], USA, in part by Kellogg	NHANES, 1999–2002	N = 1389, 1–12 y	24-h dietary recall	RTEC vs no RETC at breakfast	Energy, nutrient intake
Yeung et al, 2011 [49], USA, Centers for Disease Control and Prevention	NHANES 2003–2006	N = 7161, 1–18 y	24-h dietary recall	Folic acid enriched grain consumers (ECGP) vs ECGP + RTEC consumers vs ECGP + folic acid supplements (SUP) consumers vs ECGP+SUP+RTEC consumers	Folic-acid consumption, serum folate, red blood cell folate, serum vitamin B-12

AI: Adequate intake, EAR: estimated average requirement, FFQ: Food frequency questionnaire, NHANES: National Health and Nutrition Examination Survey, LRNI: lower reference nutrient intake, RDA: recommended dietary allowance, SBP: School Breakfast Program

^a^ Results in Table 4, S1 and S2 Tables.

**Table 2:** Characteristics prospective cohort studies assessing associations between frequency of RTEC consumption and nutrient intake and/or health outcomes.

Reference, country, sponsor	Study name	Base-line cohort, age, special characteristics	Start data collection, years of follow-up	Dietary Assessment, classification whole grain (WG)	Health outcome, number of cases, method of assessment	Exposure	Quantity	Outcome or RR (95% CI), confounders assessed
Albertson et al, 2009 [54], USA, General Mills	The Dietary Intervention Study in Children	660 children (361 boys), 8–10 y, with mean serum LDL cholesterol levels between the 80th and 98th percentile for their sex and age.	1987–1996, mean 7.5 y	Three 24-hour dietary recalls	Blood lipids and BMI, measured	RTEC	3 vs 0 servings/3 days	Total cholesterol: decreased only in boys (0.10 mmol), LDL cholesterol: decreased only in boys (0.07 mmol/l), HDL cholesterol: decreased only in girls (0.05 mmol/l), BMI: lower in boys (BMI 20.4 vs 20.1) Visit number (closely correlated with age, because all participants were aged 8 to 10 years at baseline), race, highest level of parental education, number of parents in household, intervention assignment, study site, average total energy intake, physical activity level and pubertal maturation.
Balvin et al, 2013 [51], USA, The National Institute of Diabetes and Digestive and Kidney Diseases/National Institutes of Health	A School-Based Type 2 Diabetes Prevention Program (Bienestar)	625 children, 9.1 ± 0.5 y (mean ± SD), low income	2001–2004, 3 y	Three 24-hour dietary recalls	Nutrient intake, BMI, weight and height measured	RTEC	0 times, 1 times, 2 times, 3 times/3 days	Cross-sectional data for energy and nutrient intake; BMI: decrease of 2 percentiles (-1.977 ± 0.209, estimate ± SE) for every day of RTEC consumption. Sex, ethnicity, and age (interaction effect with RTEC) and energy, total carbohydrates, and total fat (random effect).
Bazzano et al, 2005 [8], USA, General Mills	Physicians’ Health Study	17,881 men, 40–84 y	1982, 8 and 13 y	61-item FFQ; WG: BC that contained at least 25% whole grain or bran by weight	Average change in BMI and weight, self-reported	All cold BC, Whole grain BC, refined grain BC	≥ 1 serving/day vs rarely	**Body weight gain** all cold BC: 8 y: 1.07 vs 1.66 kg and 13 y: 1.81 vs 2.27 kg Whole grain BC: 8 y: 1.13 vs 1.55 kg and 13 y: 2.18 vs 1.83 kg Refined grain BC: 8 y: 1.46 vs 0.95 kg and 13 y: 2.14 vs 1.77 kg **RR BMI** all cold BC 8 y: 0.78 (0.67 to 0.91) and 13 y: 0.88 (0.76 to 1.00) Whole grain BC: 8 y: 0.83 (0.71 to 0.98) and 13 y: 0.91 (0.79 to 1.05) Refined grain BC: 8 y: 0.81 (0.64 to 1.03) and 13 y: 0.81 (0.65 to 1.01) Age, smoking, baseline BMI, alcohol, physical activity, history of hypertension, history of high cholesterol, and use of multivitamins.
Djousse et al, 2007 [56], USA, National Cancer Institute and National Heart, Lung, and Blood Institute	Physicians’ Health Study I	21,376 men, 40–86 y	1981, mean 19.6 y	61-item FFQ; WG: BC that contained at least 25% whole grain or bran by weight	Heart failure, 1018 cases, self-reported	All cold BC, whole grain BC, refined grain BC	≥ 7 vs 0 serving/week	All cold BC: 0.71 (0.60 to 0.85) Whole grain BC: 0.72 (0.59 to 0.88) Refined grain BC: 0.83 (0.58 to 1.18) Age, smoking, alcohol consumption, vegetable consumption, use of multivitamin, physical activity, history of atrial fibrillation, left ventricular hypertrophy, and valvular heart disease
Kochar et al, 2007 [55], USA, National Cancer Institute and National Heart, Lung, and Blood Institute	Physicians’ Health Study I	21,152 men, 40–86 y	1981, mean 19.1 y	61-item FFQ; WG: BC that contain at least 25% of whole grain or bran	incident DM, 1958 cases, self-reported	All cold BC, whole grain BC, refined grain BC	≥ 7 vs 0 serving/week	All cold BC: 0.69 (0.60 to 0.79) Whole grain BC: 0.60 (0.50 to 0.71) Refined grain BC: 0.95 (0.73 to 1.3) BMI, age, smoking, vitamin intake, physical activity, vegetable consumption, and alcohol intake
Kochar et al, 2012 [52], USA, National Cancer Institute and National Heart, Lung, and Blood Institute	Physicians’ Health Study I	13,368 men, 40–86 y	1981, mean 16.3 y	61-item FFQ; WG: BC that contain at least 25% of whole grain or bran	Hypertension, 7,267 cases, self-reported	All cold BC, whole grain BC, refined grain BC	≥ 7 vs 0 serving/week	All cold BC: .81 (0.75 to 0.86) Whole grain BC: 0.80 (0.74 to 0.86) Refined grain BC: 0.86 (0.74 to 1.00) Age, smoking, BMI, alcohol consumption, fruit and vegetable consumption, physical activity, and history of diabetes
Liu et al, 2000 [50], USA, National Institutes of Health	Nurses’ Health Study I	75,521 women, 38–63 y	1984, 10 y	126-item FFQ; WG: BC with 25% or greater whole-grain or bran content by weight.	Diabetes, 1,879 cases, self-reported	Whole grain BC	≥1/day vs almost never	0.66 (0.55 to 0.80). Age, BMI, physical activity, cigarette smoking, alcohol intake, family history of diabetes in a 1^st^ degree relative, use of multivitamins or vitamin E supplements, total energy intake
Wengreen et al, 2011 [53], USA, General Mills	Cache County Study on Memory, Health and Aging	N = 3634, 1564 men, 74.2 ± 6.5 y (mean ± SD), 2070 women, 75.0 ± 6.8 y; not demented and providing plausible and complete dietary data at the baseline interview	1997–1998, 2002–2003, 2006–2007, mean 11 y	142-item FFQ	cognitive function, modified Mini-mental State Examination (3MS)	RTEC	1 serving/day vs 1–6 serving/wk vs < 1 serving/wk	Cross-sectional data for energy and nutrient intake. 1–6 serving/wk group scored higher on their baseline and 11 y 3MS than the 1 serving/day and < 1 serving/wk groups (baseline 91.7, 90.6, 90.6 points respectively; 11 y 3.96, 5.13, 4.57 points respectively). Education level, BMI, IADL, level of physical activity, smoking and drinking status, history of heart attack, stroke, and diabetes, total calorie intake, and marital status

CI: Confidence interval, BC: breakfast cereals, BMI: body mass index, FFQ: food frequency questionnaire, RR: relative risk, SD: standard deviation, SE: standard error/

**Table 3:** Characteristics of (randomized) controlled trials assessing the effect of RTEC consumption on health outcomes.

Author, year (publication ref), location, sponsor	Design/ duration	Number subjects, (sex), age, BMI (kg/m^2^)	Intervention	Relevant outcomes	Results
Barkoukis et al, 2007 [64], USA, General Mills	Randomized, cross-over, acute	N = 10 (6 men), 25 ± 2 y, BMI 22.9 ± 0.7 (mean ± SEM)	136 g high DF RTEC (HF, 50 g CHO, 63,5 g DF^a^) vs 60 g low DF RTEC (LF, 50 g CHO, 2 g DF^a^), each with 300 ml of water vs 50 g glucose in 300 ml of water (for GI determination)	GI, plasma insulin: baseline and at 15, 30, 45, 60, 90 and 120 min	GI: HF: 49 ± 8, LF: 125 ± 17 Plasma insulin: 2h-AUC 50% lower after HF (7 vs 14 nmol/l·min) (estimated from graph)
Beck et al, 2009 [59], Australia, Cereal Partners Worldwide	Cross-over, acute	N = 14 overweight (7 men), 29–45 y, BMI 25–37 (range)	Corn-based RTEC with different amounts of β-glucan (BG): 39 g Control: 0 g BG 45 g low- BG dose: 2.3 g BG (LBG) 45 g mid-BG dose: 4.0 g BG (MBG) 45 g high-BG dose: 5.7 g BG (HBGO) All β -glucan from an oat bran with high β-glucan content (OatWell^™^); 45 g high-BG dose: 5.9 g BG (HBGO) containing extracted β-glucan. All with 200 ml milk and a glass of water.	Blood glucose, insulin: baseline and at 30, 60, 120, 180, 240 min, 4-question appetite VAS at same time points, food intake at buffet lunch served 4 h after RTEC consumption	Glucose: not different Insulin: 2h-AUC but not 4h-AUC lower after MGB, HBGO and HBGX than control Combined appetite VAS: less appetite after all BG-RTEC compared to control, Food intake at lunch: not significantly different
Bell et al, 1990 [72], USA, General Mills	double-blind, placebo-controlled, randomized, 3 x 6 wk	N = 58 men with hyper-cholesterolemia, 24–69 y (range)	Prudent diet with 3 different RTEC a 57 g: Control RTEC (0.5 g DF, 0 g soluble DF, 0.5 g insoluble DF) Pectin-enriched RTEC (13.6 g DF, 6.1 g soluble DF, 7.5 g insoluble DF) Psyllium-enriched RTEC (10.0 g DF, 5.8 g soluble DF, 4.2 g insoluble DF)	Blood lipids	Psyllium-enriched cereal:additional reduction of 0.34 mmol/l (5.9%) of total cholesterol and 0.23 mmol/l (5.7%) LDL over diet-only values compared to baseline, changes different from control.
Brighenti et al, 1999 [69], Italy, National Research Council of Italy	Cross-over, 2 x 4 wk afterwards 4 wk washout	N = 12 men, 23.3 ± 0.5 y, BMI 25.7 ± 1.2 (mean ± SEM)	Period 1: 50 g/day control RTEC Period 2: 50 g/day RTEC with 9 g of inulin Wash-out period: habitual diet	Total cholesterol, LDL, HDL, TAG, intestinal habits, microbiota (selective growth media)	Blood lipids: Inulin RTEC: total cholesterol lower than basal (- 0.35 mmol/l, 8.25%) but not lower than placebo, TAG lower than basal and placebo (both—0.23 mmol/l, 27.4%) Intestinal habits: no differences Microbiota: Compared to baseline: Total facultative anaerobes lower after inulin RTEC (9.29 vs 8.52 log CFU/g dry weight); Post-treatment inulin RTEC vs control: Bifidobacteria higher (10.66 vs 10.99) only after correction for total anaerobes; Total anaerobes, Bacteroides, Clostridia, coliforms: no difference
Carvalho-Wells et al, 2010 [58], UK, Cereal Partners UK	Double-blind, randomized placebo controlled, cross-over, 2 x 3 wk with 3 wk washout and 2 wk run in	N = 32 (11 men), 20–51 y, BMI 20–30 (range)	48 g/day whole grain RTEC (WG, 29.6% whole grain maize, 6.8 g DF) vs 48 g/day non-whole grain RTEC (nonWG, 0.4 g DF)	Primary: Microbiota (FISH); secondary: bowel habits, blood lipids	Microbiota: compared to baseline: increase in *Bifidobacterium* spp. after WG (9.53 vs 9.81 log_10_ bacteria/g wet weight feces), increase in *Atopobium* cluster spp. after WG (9.63 vs 9.94) and non-WG (9.64 vs 9.95); post-treatment WG compared to non-WG: borderline significant higher *Bifidobacterium* spp (9.9 vs 9.6, p = 0.0561); *Lactobacillus/Enterococcus*, *Bacteroides* spp., *Clostridium histolyticum* subgroup, C. *perfringens/ histolyticum* subgroup and total bacterial numbers: no difference Bowel habits and blood lipids: no differences
Costabile et al, 2008 [10], UK, Cereal Partners UK	Double-blind, randomised, cross-over, 2 x 3 wk and 2 wk washout	N = 31 (15 men), 20–42 y, BMI 20–30 (range)	48 g/day whole grain wheat RTEC (WG, 5.7 g DF) vs 48 g/day wheat bran RTEC (WB, 13 g DF)	Primary: Microbiota (FISH); secondary: bowel habits, blood lipids	Microbiota: compared to baseline: increase in *Bifidobacterium* spp. after WG (8.5 vs 9.3 log_10_ cells/g feces); post-treatment WG compared to WB: higher *Bifidobacterium* spp (9.3 vs 8.8) and *Lactobacillus/Enterococci* (8.7 vs 8.4); Total bacteria, *Bacteroides* spp., *Eubacterium rectale* group, Atopobium spp., *Clostridium histolyticum/perfringens* gp.: no difference Bowel habits: Stool frequency: higher during ingestion of WB compared with WG;stool consistency: greater proportion of stools described as formed after WG ingestion; increase in soft stools and flatulence after WB ingestion Blood lipids: no differences
Fairchild et al, 1996 [70], UK, British Diabetic Association	Randomized cross-over, acute	N = 10 (3 men), 22.6 ± 1,2 y, BMI 22.3 ± 0,5 (mean ± SEM)	64 g wheat flakes (control, 50 g CHO, 1 g soluble NSP, 3 g insoluble NSP) vs 70 g Guar wheat flakes (50 g CHO, 5.5 g soluble NSP, 3.6 insoluble NSP). All with 201 g milk and 209 g orange juice	Blood glucose, insulin: baseline and at 15, 30, 45, 60, 90, 120, 150, 210 and 240 min	Glucose: 1h and 2h-AUC 42% and 47% respectively lower after guar wheat flakes Insulin: 1h, 2h and 4h-AUC 28%, 34% and 35% respectively lower after guar wheat flakes
Hamedani et al, 2009 [61], Canada, General Mills	repeated-measures cross-over, acute	N = 32 (16 men), 20–26 y, BMI 20.5–24.5 (range)	60 g high DF RTEC (HF, 28 g total DF, 2 g soluble DF) vs 60 g low DF RTEC (LF, 1.5 g total DF, 1.2 g soluble DF). All with 250 mL milk and 250 mL water. Total test meal: HF: 120 kcal, LF: 217 kcal	VAS every 15 min during the first and last hour and every 30 min during the second and third hour: average appetite score from desire to eat, hunger, fullness, prospective food consumption, energy intake at ad libitum lunch 170 min after breakfast consumption	Average appetite score: no difference Energy intake not different after lunch Cumulative energy intake (breakfast and lunch) lower after HF (1329.9 ± 57.1 vs 1422.4 ± 5.6 kcal)
Hlebowicz et al, 2007 [65], Sweden, not stated	randomized cross-over blinded trial, acute	N = 12 (6 men), 28 ± 4 y, BMI 22 ± 2 (mean ± SEM)	50 g All Bran (7.5 g DF, 163 kcal) vs 50 g wholemeal oat flakes (4 g DF, 185 kcal) vs 50 g cornflakes (1.5 g DF, 185 kcal). All with 300 g sour milk	Satiety: VAS at 0, 20, 30, 40, 60, 80, 100 and 120 min	Satiety: no difference
Hlebowicz et al, 2008 [76], Sweden, Skanemejerier	Randomized cross-over trial, acute	N = 12 (8 men), 27 ± 5 y, BMI 22 ± 3 (mean ± SEM)	26.5 g muesli with 24.5 g oat b-glucan flakes (9 g DF, 4 g b-glucan, 72 kcal) vs 26.5 g muesli with 17.5 g cornflakes (0.5 g DF, 72 kcal). All with 200 g yoghurt and 200 ml of water	Satiety: VAS at 15 and 90 min	Satiety: no difference
Ingwersen et al, 2007 [75], UK, in part by Cambridge Laboratories	Balanced cross-over, acute	N = 64 (26 boys), 6–11 y (range)	35 g High GI (77) cereal vs 35 g low GI (42) cereal. All with 125 ml milk	Attention, memory using the Cognitive Drug Research Computerized Assessment Battery (25 min duration)	Secondary memory: better performance after low GI cereal (-30.68) vs high GI cereal (-47.18) Accuracy of attention: decline in performance at 11.40 a.m. higher after high GI cereal Speed of attention and memory, working memory: no effect
Kleemola et al, 1999 [11], Finland, Kellogg	Open, randomized, cross-over, 2 x 6 wk and 6 wk washout	N = 209 (95 men), 29–71 y (range), Plasma cholesterol ≥ 5 mmol/l	60 g/day RTEC for women and 80 g/day RTEC for men vs habitual Finnish breakfast (control)	Primary: total and saturated fat intake, serum cholesterol Secondary: body weight	Difference in change between RTEC group and control: Total cholesterol: 0.16 mmol/l lower HDL: 0.05 mmol/l lower LDL: not measured Total and saturated fat intake: 5.5 and 2.5 energy % respectively lower Body weight: no changes
Lafond et al, 2015 [57], USA, Kellogg	randomized, double-blind, placebo-controlled, cross-over, acute	Trial 1: n = 30 overweight women, 22.5 ± 0.6 y, BMI 27.0 ± 0.3 (mean ± SEM) Trial 2: n = 36 overweight women, 24.3 ± 0.5 y,BMI 27.4 ± 0.3	Trial 1: 100 g low DF RTEC (LF, 461 kcal, 4 g DF) with 180 g milk vs 100 g high DF RTEC with enzyme hydrolyzed and purified wheat-bran arabinoxylan extract (HF-AXOS, 345 kcal, 19 g DF) with 200 g milk vs 100 g high DF RTEC with unhydrolyzed flax seed fiber (HF-FLAX, 345 kcal, 19 g DF) with 200 g milk Trial 2: As trial 1 and 70 g LF RTEC (LF-iso: isocaloric to the HF meals, 3 g DF) and 170 g milk	Trial 1 and 2: Appetite: desire to eat, hunger, fullness, prospective consumption. VAS at t = - 15, - 5, 15, 30, 45, 60, 75, 90, 105, 120, 135, 150, 165, 180, 195, 210, 225, 240, and 270 min; Food intake ad libitum lunch at 240 min	Trial 1: VAS, energy intake at lunch, cumulative energy intakes (breakfast and lunch): no differences Trial 2: VAS, energy intake at lunch: no differences;Cumulative energy intake: lower after LF-iso, HF-AXOS and HF-FLAX (899 ± 38, 907 ± 37, 894 ± 36 kcal respectively) than after LF (994 ± 37 kcal)
Levine et al, 1989 [73], USA, Veterans administration research funds and the national Institute of Drug Abuse grant	Randomized, parallel, acute	Experiment 1: N = 14 (sex not given), 24–59 y, BMI 22 ± 0.5 (mean ± SEM) Experiment 2: N = 19 (sex not given), 24–55 y, BMI 24 ± 0.9	Experiment 1: 5 breakfasts a 57 g: Fiber One (22 g DF, 120 kcal), All Bran (20 g DF, 246 kcal), Bran Chex (10 g DF, 180 kcal), Shredded Wheat (6 g DF, 180 kcal), Post Toasties (0 g DF, 221 kcal), all with 240 ml milk and 120 ml orange juice Experiment 2: 2 breakfasts a 57 g: Fiber One (22 g DF, 120 kcal), Post Toasties (0 g DF, 221 kcal), all with 240 ml milk and 120 ml orange juice	Experiment 1 and 2: Energy intake at ad libitum lunch 3.5 h after breakfast;Questionnaire: Degree of hunger before lunch	Experiment 1: Lower cumulative energy intake (breakfast and lunch) after All Bran and Fiber One compared to Post Toasties (1185 ± 87 and 1176 ± 67 vs 1324 ± 87 kcal respectively); Degree of hunger: lower after All Bran than after Post Toasties and Bran Chex Experiment 2: Lower energy intake at lunch and cumulative energy intake after Fiber One (≈ 100 and 200 kcal respectively, estimated from graph); Degree of hunger: no difference
Lioger et al, 2009 [60], France, Kellogg	Randomized, cross-over, acute	N = 11 men, 18–30 y (range), BMI 21.4 ± 0.7 (mean ± SEM)	70 g Standard wheat flakes (SWF, 39 g starch, 10.1 g sugars, 8 g DF) vs 77 g modified WF (MWF, 45.8 g starch, 5.1 g sugars, 10.2 g DF) vs 89.3 g of white-wheat bread (WWB, 48.2 g starch, 1.7 g sugars, 3.1 g DF); Modification in MWF: 1/3 of whole wheat flour was fermented, the steam cooking step omitted and the sucrose content half of that of SWF (5.2 vs 11.6%)	Blood glucose, insulin: baseline and at 15, 30, 45, 60, 90, 120, 150, 180 min, VAS feeling of hunger: baseline and at 30, 60, 90, 120, 150, 180 min, GI and insulinemic index (II) calculated	GI: no differences II: 90 min and 180 min of MWF lower than that of SWF (78 ± 6 vs 98 ± 8 and 85 ± 5 vs 96 ± 5 respectively) Hunger feeling: 120, 150 and 180 min after MWF lower than after WWB and SWF
Roberts et al, 1994 [71], Australia, Kellogg	Double-blind, cross-over, 2 x 6 wk, no washout	N = 81 men with hyper-cholesterolemia, 31–69 y, BMI 19–34 (range)	Low saturated fat diet with 60 g/day wheat/wheat bran cereals (control) (12.6 g DF, 1.6 g soluble DF, 11.0 g insoluble DF) vs 50 g/day Psyllium/oat/barley cereal (15.2 g DF, 11.9 soluble DF (86% from psyllium), 3.3 insoluble DF)	Blood lipids	Psyllium/oat/barley cereal: Reduction of 0.23 mmol/l (3.5%) total cholesterol, 0.26 mmol/l (5.7%) LDL and 0.04 mmol/l (3.3%) HDL compared to baseline. Changes different from control values.
Rosado et al, 2008 [62], Mexico, in part by Kellogg	Randomized controlled, parallel, 12 wk	N = 178 (86 boys), 6–12 y (range), BMI ≈ 24	Group 1: 1 serving of 33 ± 7 g of RTEC at breakfast Group 2: 2 servings of same RTEC at breakfast and dinner Group 3: 1 serving of RTEC and healthy eating education for children and mothers Group 4: No treatments	Anthropometry, body composition and blood lipids at the beginning and end of the study	Body weight: increased in group 1, 2 and 4, decreased only in group 3: - 1.1 kg, different to all other groups BMI change: Group 3: - 0.9, different to all other groups Body fat change: Group 3: - 0.8%, different to group 1 and 4 Blood lipids^b^: Group 3: TG: - 20.7 mg/dl compared to baseline; VLDL: - 3.8 mg/dl compared to baseline; HDL: + 6.6 mg/dl compared to baseline and different to group 1 and 4
Samra et al, 2007 [12], Canada, General Mills	Randomized, repeated-measures cross-over, acute	Experiment 1: N = 16 men, Experiment 2: N = 15 men, 20–35 y, BMI: 20–27 (range)	Experiment 1: 71 g high DF RTEC (HF, 33 g DF) and 140 g water vs 30 g low DF RTEC (LF, 1 g DF) and 200 g water vs 76 g white bread (WB, 0 g DF) and 160 g water. All with 250 ml milk, all with same calories (± 285 kcal), macro-nutrients, weight and volume, 500 ml water as control Experiment 2: same treatments except water and except that WB was served cut into 2-cm 3 pieces and dipped in the milk to be eaten with a spoon (similar to the cereals)	Experiment 1: energy intake at ad libitum meal 75 min after breakfast; VAS at baseline, 30, 45, 60, 75 and 90 min: average appetite score from desire to eat, hunger, fullness, prospective consumption; Experiment 2: VAS as Experiment 1	Experiment 1: Energy intake: lower after HF cereal and WB than after LF cereal and water(≈940 kcal HF vs ≈1100 kcal LF, estimated from graph). Average appetite: HF cereal lowest AUC (-1792 ± 438.7 mm·min), followed by the LF cereal (-1224 ± 334.6 mm·min), WB (-766 ± 342.8 mm·min), and water (310 ± 141.3 mm·min) Experiment 2: Average appetite scores not different
Schenk et al, 2003 [67], USA, Quaker Oats	Randomized, cross-over, acute	N = 6 men, 27.8 ± 1.5 y (mean ± SEM), BMI 22.8 ± 0.24	119.2 g high DF RTEC (BC, 50 g available CHO, 15.4 g protein, 38.5 g total DF, 3 g soluble DF) vs 60.9 g low DF flakes (CF, 50 g available CHO, 4.3 g protein, 1.7 g total DF, 1.4 g soluble DF)	Blood glucose, insulin: baseline and at 20, 30, 60, 90, 120, 150, and 180 min; Glucose kinetics: rate of appearance of exogenous glucose (Ra_gluc_), glucose uptake from the blood by tissues (Rd_gluc_), glucose clearance rate (GCR)	Glucose: 3 h-AUC 55% lower after BC (192.5 ± 3 8.4 compared with 85.7 ± 12.1 mmol·min/L) GI of BC: 59% lower (131.5 ± 33.0 vs 54.5 ± 7.2) Insulin: 125% higher after BC during 0–30 min (288.7 ± 71.0 vs 128.4 ± 42.9 uU·min/mL) Ra_gluc_: No differences Rd_gluc_: 31% higher after BC during 30–60 min only (28.7 ± 3.1 vs 21.9 ± 3.1 mol/kg·min) GCR: after BC 54% higher during 30–60 min (5.3 ± 0.8 vs 3.5 ± 0.6 ml/kg·min)
Schorah et al, 1998 [77], UK, Kellogg	randomized double-blind placebo-controlled, 24 wk	N = 94 (47 men) non-consumers of vitamin supplements and RTECControl group: N = 30, 36–65 y Group 1: N = 33, 32–59 y Group 2: N = 31, 36–58 y (interquartile ranges, the 25^th^ and 75^th^ percentiles)	Control group (CG): 30 g unfortified RTEC Group 1: 30 g RTEC with ca 200 ug folic acid Group 2: 30 g RTEC with ca 200 ug folic acid and other vitamin fortification	Plasma tHcy, serum and red cell folate, baseline and at 4, 8 and 24 wk	Changes of serum folate and tHcy from baseline different in group 1 and group 2 (all time points). Changes in group 1 and 2 different from those in CG for serum folate at 4, 24 wk and 4, 8, 24 wk respectively, for tHcy at 4, 8, 24 wk and 8, 24 wk respectively for red cell folate at 24 wk and 4 and 24 wk respectively.
Smith et al, 2008 [63], Australia, not stated	Randomized, parallel, acute	N = 38 (19 men), 15.6 ± 0.9 y (mean ± SD)	30 g high GI (77) RTEC vs 30 g low GI (30) RTEC, all with 125 ml milk	Modified California Verbal Learning Test (CVLT) with secondary task, Bond-ladder scale for mood and affect	Modified CVLT: no effect immediate, short- and long-delay recall Remembering/forgetting indices (post-hoc-test): more items remembered at long vs short delay after high GI cereals and at long delay after high vs low GI cereals Bond-ladder scale: no effect
Tucker et al, 2004 [66], USA, Kellogg	double-blind, randomized, controlled, parallel, 14 wk	N = 189 (84 men) no vitamin supplements and/or highly fortified breakfast cereals Treatment group: n = 93, 65.4 ± 9.3 y (mean ± SD), BMI 26.2 ± 3.9, Control group: n = 96, 65.1 ± 8.8 y, BMI 26.9 ± 5.0	1 cup (0.24 l) breakfast cereal fortified with 440 μg folic acid, 1.8 mg vitamin B 6, and 4.8 μg vitamin B 12 or an identical cereal without the addition of these vitamins.	Plasma folate, vitamin B 12, B 6 (PLP), fasting tHcy: baseline and posttreatment (mean 12 and 14 wk), tHcy 2 h after methionine-load test: baseline and at 14 wk	Treatment group: Folate: increase from 25 to 32 nmol/L, B-12: increase from ≈296 to 354 pmol/L, PLP: increase from 52 to 82 nmol/L, Fasting tHcy: decrease from 7.9 to 7.5 μmo/L, Post-methionine load tHcy: decrease from 22.7 to 21.3 μmol/L Control group: PLP: decrease from 46 to 42 nmol/L
Venn et al, 2002 [68], New Zealand, in part by Kellogg	Double-blind, randomized placebo-controlled, 4 wk	N = 70 (37 men), fasting plasma tHcy ≥ 10 μmol/l Control group: n = 14, 60 ± 14.5 y (arithmetic mean ± SD) Group 1: n = 19, 58 ± 14.8 y Group 2: n = 22, 61 ± 11.2 y Group 3: n = 15, 52± 12.8 y	Control group (CG): 20 g unfortified RTEC, Group 1, 2, 3: 20 g RTEC with 100, 200, 300 μg folic acid/serving respectively	Plasma tHcy, serum folic acid, baseline and at 4 wk	Compared to CG serum folate increased in parallel with increasing supplemental folic acid by 28, 60 and 79% for groups 1, 2 and 3 respectively
Wolever et al, 2004 [74], Canada, General Mills	Randomized, cross over, acute	N = 42 hyper-insulinemic men, 41 ± 2 y (mean ± SEM), BMI 29 ± 0.5 N = 37 healthy men, 43 ± 3 ys, BMI 26 ± 0.6	77.2 g high DF RTEC (HF) vs 30.0 g low DF RTEC (LF). Allwith 250 ml of milk and 250 ml of water. Total test meal: HF: 36.8 g available CHO, 36.7 g DF (35.8 g insoluble DF); LF: 36.8 g available CHO, 0.8 g of DF (0.5 g insoluble).	Blood glucose and insulin, baseline and at 15, 30, 45, 60, 90, 120 min	Healthy control: 2h-AUC glucose: 14% lower after HF RTEC (112 ± 1 0 vs 130 ± 10 mmol·min/l); 2h-AUC insulin: not different (11.5 ± 0.9 vs 11.9 ± 1.2 nmol·min/l). Hyperinsulinemic subjects: 2h-AUC glucose: 21.5% lower after HF RTEC (102 ± 10 vs 130 ± 11 mmol·min/l); 2h AUC insulin: 14% lower after HF RTEC (20.8 ± 2.0 vs 24.2 ± 2.2 nmol·min/l)

AUC: area under the curve, BMI: body mass index, CHO: carbohydrates, DF: dietary fiber, FISH: fluorescence in situ hybridization, GI: glycemic index, Homocysteine: tHcy, NSP: non-starch polysaccharides, VAS: visual analog scale

^a^DF content derived from product information in internet,

^b^data not adjusted for initial value, gender, school random effect and significant interactions.

### Nutritional benefits—associations and effects of interventions

One prospective [54] and 31 cross-sectional studies in different countries (15 in the USA [7;23;25;27–29;31;32;34;36;42–44;47;48], three in Canada [22;24;46], two each in Spain [33;41], Ireland [37;39], Australia [26;45] and France [9;38] and one each in Scotland [40], Cyprus [20], Greece [35], Malaysia [21] and Guatemala [30]) investigated the association between RTEC consumption and daily nutrient intake. From two prospective studies [51;53] baseline data concerning RTEC consumption and daily nutrient intake were also used. One cross-sectional study assessed the impact of RTEC consumption on micronutrient status only [49].

23 studies included only children/adolescents [7;20–23;26;27;29–31;35;36;38–41;43–45;47–49;51], eight studies only adults [9;24;25;28;34;37;38;53], one study children and adults [42] and in three studies the age-range comprised children/adolescents and adults [32;33;46] (categorized in “adult” studies). In cross-sectional studies frequency of RTEC consumption was mainly assessed with single and repeated 24-h dietary recalls ([20–24;27–35;42;44;47;49] and [9;26;45] respectively) (Table 1). In addition, food diaries of 14 [7;25;36], 7 [37;43;46] and 3 days [78] as well as 5 –and 7 day weighted food records were used [40;41]. Two studies applied the dietary history method [38;39]. Due to the variation in registration of food intake, comparisons of high or low frequency of RTEC consumption in these studies varies from 1 serving/day vs none to 7/14 day vs < 2/14 days. RTEC consumption at breakfast only was monitored in 20 studies [20–24;26;28;29;31;32;34;38;40;41;43;45;47;48;51;54]. RTEC consumption during the whole day was assessed in 14 studies of which in four it was demonstrated that most of the RTEC were consumed at breakfast (Ireland: 91% [37], Spain: 67% [33], France: 89% [9], Guatemala: 93.2% [30]) and in three studies that a high percentage of the population was eating RTEC at breakfast (USA: 63% of 10 y olds and 65% of young adults [42] and 87,3% of 4–12 y old children [44], Greece: 60% of boys and 58% of girls [35]). As RTEC, based on these numbers, are predominately eaten at breakfast, all the studies were included. One RCT in adults examined the impact of substituting a traditional breakfast by RTEC on daily macronutrient consumption [11].

#### Associations of RTEC consumption with daily intake of energy, macronutrients, cholesterol, dietary fiber (DF) and sodium

For summarizing cross-sectional data about the association between RTEC consumption and daily intake of energy, macronutrients, cholesterol, DF and sodium in children/adolescents, 33 data sets from 24 studies were available. More data sets per study were available when the investigators reported their results per sex and/or in several age groups (S1 Table and Table 4). 18 of these studies were (in part) funded by food industry. Higher frequency (approximately ≥ 5 serving/week) of RTEC consumption in children/adolescents was associated with higher intake of DF, carbohydrates and total sugars in 75%, 65% and 63% of the data sets respectively and with lower intake of cholesterol and fat, expressed as total amount and as energy percentage in 83%, 50%, and 60% of the data sets respectively. Energy, saturated fat, sodium and protein intake was not associated with RTEC consumption in most data sets (in 77%, 75%, 81% and 86% respectively). Associations were similar in the 6 studies (9 data sets) [20;21;41;43;48;51] with no food-industry related funding, except that total amount of fat was only reduced in 22% of the 9 data sets and dietary fiber intake was only higher in 25% of the 4 data sets in which it was measured. In the other data sets (78% and 75%) no difference of fat and dietary fiber intake was reported.

**Table 4:** Number and percentages of studies reporting higher, lower or equal daily consumption of energy and nutrients of frequent versus low/no RTEC consumers.

**Children/adolescents (summary of 33 data sets from 24 studies)**									
	**Energy**	**Dietary fat, total amount**	**Saturated fat, total amount**	**Cholesterol, total amount**	**Carbohydrates, total amount**	**Total sugars, total amount**	**Dietary fiber, total amount**	**Sodium, total amount**	**Protein, total amount**
Higher intake	5 (19%)	2 (8%)	0	0	13 (65%)	15 (63%)	18 (75%)	1 (5%)	2 (9%)
Lower intake	1 (4%)	12 (50%)	3 (25%)	20 (83%)	1 (5%)	0	0	3 (14%)	1 (5%)
Equal intake	20 (77%)	10 (42%)	9 (75%)	4 (17%)	6 (30%)	9 (37%)	6 (25%)	17 (81%)	18 (86%)
Not assessed	7	9	21	9	13	9	9	12	12
		**Dietary fat, % of energy**	**Saturated fat, % of energy**		**Carbohydrates, % of energy**	**Total sugars, % of energy**	**Whole grain, total amount**		**Protein, % of energy**
Higher intake		3 (20%)	0		7 (58%)	3 (100%)	5 (100%)		1 (10%)
Lower intake		9 (60%)	1 (14%)		0	0	0		1 (10%)
Equal intake		3 (20%)	6 (86%)		5 (42%)	0	0		8 (80%)
Not assessed		18	26		21	30	28		23
**Adults (summary of 16 data sets from 12 studies)**									
	**Energy**	**Dietary fat, total amount**	**Saturated fat, total amount**	**Cholesterol, total amount**	**Carbohydrates, total amount**	**Total sugars, total amount**	**Dietary fiber, total amount**	**Sodium, total amount**	**Protein, total amount**
Higher intake	5 (38%)	0	0	0	9 (100%)	7 (100%)	13 (93%)	2 (33%)	2 (25%)
Lower intake	0	3 (30%)	4 (57%)	4 (57%)	0	0	0	0	1 (13%)
Equal intake	8 (62%)	7 (70%)	3 (43%)	3 (43%)	0	0	1 (7%)	4 (67%)	5 (62%)
Not assessed	3	6	9	9	7	9	2	10	8
		**Dietary fat, % of energy**	**Saturated fat, % of energy**		**Carbohydrates, % of energy**	**Total sugars, % of energy**	**Whole grain, total amount**		**Protein, % of energy**
Higher intake		0	0		10 (100%)	3 (100%)	2 (100%)		0
Lower intake		10 (100%)	1 (33%)		0	0	0		3 (43%)
Equal intake		0	2 (67%)		0	0	0		4 (57%)
Not assessed		6	13		6	13	14		9

For summarizing the results in adults 16 data sets from 12 studies were available (S1 Table and Table 4). One of these studies [28] received no food-industry related funding. Higher frequency (approximately ≥ 5 serving/week) of RTEC consumption in adults was associated with higher intake of DF, carbohydrates and total sugars in 93%, 100% and 100% of the data sets respectively and lower intake of fat expressed as energy percentage (in 100% of data sets) but not if expressed as total amount. The associations of RTEC consumption with saturated fat and cholesterol intake were not consistent, whereas most data sets (62%) did not show an association with energy and protein intake.

One study (funded by food industry) investigated the association between RTEC consumption and daily intake of energy, macronutrients, cholesterol, DF and sodium prospectively [54] (Table 2). In secondary analyses of a RCT, 8–10 y old children were followed for 7.5 y Higher frequency (3 vs 0 serving/3 days) of RTEC consumption at breakfast was associated in girls and boys with a higher percentage of energy intake from carbohydrates and protein as well as a lower percentage from total and saturated fats. In addition, an association with higher intake of DF and lower intake of cholesterol was found whereas energy and sodium intake was not related. For boys, but not for girls, RTEC consumption was associated with higher intake of total sugars.

#### Associations of RTEC consumption with the percentage of populations receiving inadequate amounts of vitamins and minerals

17 studies (of which one [20] was not funded by food industry) investigated the association of frequency of RTEC consumption with the proportion of the population receiving inadequate vitamins and minerals, of which ten studies were conducted in children/adolescents [7;20;22;23;26;27;36;39;44;47], five studies in adults [24;25;33;37;46] and two in both categories [32;42] (Table 1).

Inadequate micronutrient intake was defined as “below the estimated average requirement (EAR)” in ten studies [22–25;27;32;36;37;44;46], as “receiving less than two-thirds of the recommended dietary allowance (RDA)” in three studies [20;33;42] and as “consuming less than 100% of RDA, “consuming less than 80% of RDA”, “probability of not achieving 100% of EAR “and “percentage who did not achieve LRNI” in one study each [7;26;39;47]. The EAR is defined as the intake adequate for 50% of the population, the RDA is the average daily dietary intake level that is sufficient to meet the nutrient requirement of nearly all (97 to 98 percent) healthy individuals and the LRNI is the amount estimated to meet the needs of 2.5% of the population with the lowest requirements.

The prevalence of inadequate vitamin and mineral intake by breakfast group (lowest vs highest RTEC consumption) for 14 micronutrients is given in S2 Table. 19 data sets were available for children/adolescents and 11 for adults. In 15 data sets it was assessed whether the prevalence of inadequacy between low and high RTEC consumers is significantly different.

Combining these results, significant reductions of prevalence of inadequacy associated with RTEC consumption were observed for all vitamins and minerals. Prevalence of inadequacy as well as magnitude of reduction varied depending on country, age group, sex and method of assessment (S2 Table).

To assess the nutrients for which the prevalence of inadequacy was reduced the most, those nutrients were scored which had the four highest reductions of inadequacy. In case that an equal percentage of reduction was observed for more micronutrients, all micronutrients were scored, thus more micronutrients per reduction level were possible (S2 Table). To reduce imbalance due to limited assessment of micronutrients, data of studies were excluded which reported less than eight micronutrients, resulting in 11 data sets for children and eight for adults. The scored micronutrients of different populations were then combined: data of adults in which significance was assessed (7 data sets), data of children in which significance was assessed (6 data sets), all adult and all children/adolescent data sets.

When using only data sets that assessed significance, in children/adolescents as well as in adults, reductions of prevalence of inadequacy due to RTEC consumption were highest for vitamin A (range: 7–21% and 5–37% respectively), calcium (17–39% and 6–40% respectively), folate (5–28% and 7–50% respectively), magnesium (7–11% and 4–26% respectively) and zinc (9% and 19–37% respectively). In adults, high reductions were also seen for vitamin B 6 (7–55%) and C (6–21%).

When combining all data sets, for children/adolescents as well as for adults consistently the greatest reductions of prevalence of inadequacy was observed for vitamin A (range: 7–28% and 5–37% respectively), calcium (17–39% and 6–43% respectively), folate (5–50% and 7–50% respectively), vitamin B 6 (31–37% and 7–55% respectively), magnesium (7–11% and 4–26% respectively) and zinc (9–15% and 19–37% respectively).

#### Associations of RTEC consumption with micronutrient status

In two cross-sectional studies (of which one was funded by food industry [38]) micronutrient status (12 vitamins and minerals) was measured in populations with and without RTEC consumption [38;41] (Table 1). In Spanish and/or French children and adolescents consumption of RTEC was associated with higher plasma concentrations of vitamin A (0.10 μmol/l [41]), β-carotene (0.21 μmol/l [38]), serum folate (4.1 nmol/l [41]) and lower erythrocyte glutathione reductase (EGR, 0.07 [38;41]) which indicates a better riboflavin status. In Spanish adults, a better thiamine and riboflavin status (erythrocyte transketolase 0.03, EGR 0.06) as well as higher β-carotene (0.25 μmol/l) and serum folate concentrations (30 μg/l) were found in the RTEC group [38].

Another study (no industrial funding) investigated the contribution of consumption of folic-acid enriched RTEC to folate and vitamin B 12 status in US children and adolescents [49] (Table 1). Higher folate and vitamin B 12 concentrations were associated with consumption of enriched RTEC. However, only a very low percentage of this population (< 0.5%) had folate deficiency or low vitamin B-12 status, probably due to consumption of other grain products which are mandatory enriched with folate since 1996. The percentage of persons with marginally low folate and vitamin B-12 status decreased from 3.4% to 1.7% and from 9.6 to 6.6% respectively [49] due to additional consumption of RTEC.

#### The effect of RTEC consumption on macronutrient and DF intake

In healthy adults substituting the habitual breakfast with RTEC (60 g for women/80 g for men) resulted in a higher percentage of energy from carbohydrates and a lower percentage from total and saturated fat [11] (Table 3) in this study funded by food industry. Intake of energy, protein and cholesterol stayed the same. No effect on DF intake was observed, but the RTEC administered were relatively low in DF, providing 3 and 4 g DF/day for women and men respectively.

### Health benefits—associations and effects of interventions

#### Associations of RTEC consumption with risk factors for cardiovascular diseases

One prospective study investigated the association of consumption of whole grain vs refined grain RTEC with incident heart failure in a large cohort of male physicians [56] (Table 2). Decreased risk of heart failure was found for frequent consumers of whole grain RTEC (HR: 0.78 (95% CI 0.64–0.96) for 2–6 servings/wk; HR: 0.72 (95% CI 0.55–0.88) for ≥ 7 servings/wk) but not for those consuming refined RTEC.

Another prospective study investigated the association of consumption of whole grain vs refined grain RTEC with incident hypertension in a large cohort of male physicians [52] (Table 2). Decreased risk of hypertension was clearly demonstrated for participants with a high consumption of whole grain RTEC (HR: 0.87 (0.81–0.94) for 2–6 servings/wk, HR: 0.80 (0.74–0.86) for ≥ 7 servings/wk), whereas the associations with consumption of refined RTEC was weak and not significant in all groups (HR: 0.86 (95% CI 0.76–0.98) for 2–6 servings/wk, HR: 0.86 (95% CI0.74–1.00) for ≥ 7 servings/wk).

One prospective study [54] (Table 2) investigated the association between low and high RTEC consumption (0 vs 3 servings/3 days) on blood lipids in a group of 660 children, aged 8–10 years at baseline and with serum LDL cholesterol levels between the 80th and the 98th percentile for sex and age. In the high RTEC group total cholesterol was lower in boys (0.10 mmol/l) but not in girls. LDL cholesterol was lower in boys (0.07 mmol/l) but in girls lower HDL cholesterol (0.05 mmol/l) was observed.

The two prospective studies reporting decreased risk of heart failure and hypertension were not funded by food industry [56; 52], whereas the last described prospective study [54] which was funded by food industry showed mixed results on blood lipids in children.

#### Effects of RTEC consumption on risk factors for cardiovascular diseases

Two RCTs, one in children (overweight or at risk of overweight 6- to 12-year-old Mexican children [62]) and one in Finnish adults [11] investigated whether increased consumption of RTEC results in a reduction of blood lipids (Table 3).

Twelve weeks of RTEC consumption (≈ 33 g, different corn and rice based types) resulted in an increase in HDL concentrations (as compared to baseline and the control group) when combined with nutritional education [62] in children. The changes of other lipid parameters, however, were not different.

In adults with serum cholesterol concentrations above 5.0 mmol/l, six weeks of consumption of RTEC (60 g for women/80 g for men) mainly in the morning instead of the habitual Finnish breakfast resulted in a reduction in total cholesterol by 2.5% (0.16 mmol/l) which was partly due to a reduction in HDL cholesterol (LDL was not measured) [11]. Intake of saturated fat and total fat was decreased by 2.5 and 5.5 energy% respectively.

Both studies were (partially) funded by the food industry and showed mixed results on the parameters investigated.

The effect of RTEC enriched with different types of DF on blood lipids was investigated in five RCTs [10;58;69;71;72] (Table 3).

Six week consumption of DF-enriched RTEC providing 5.8 or 11.9 g soluble fiber/day consistently lowered total cholesterol and LDL (by 5.9 or 3,5% and 5.7 or 5.7% respectively) in persons with hypercholesterolemia consuming a low-fat diet [71;72]. The effective soluble fiber was mainly derived from psyllium whereas soluble fiber from pectin [72] or wheat bran [71] did not have a significant cholesterol lowering effect.

Three other, more short-term (3–4 wk), RCTs in healthy volunteers [10;58;69] investigated the effect of RTEC rich in various DF (inulin-enriched vs inulin-free, whole grain wheat vs wheat bran-based, whole grain maize vs refined maize) on various lipid parameters. No effect on total and HDL cholesterol was found, only the inulin-enriched RTEC (9 g inulin/day) was able to reduce LDL cholesterol compared to baseline (by 0.35 mmol/l, 8.3%) but not to control. However, concentrations of triacylglycerols were reduced compared to baseline and to control (by 0.23 mmol/l, 27.4%) [69].

Four RCTS were industrial funded, of which two [71;72] showed positive effects on blood lipids and two no effects [10; 58]. Another RCT [69], without industrial funding, showed mixed effects.

Three RCTs [66;68;77] investigated the effect of folate-fortified RTEC on plasma homocysteine (tHcy) (Table 3). It was found that cereals fortified with 200 μg per portion could increase plasma folate concentrations by about 12 nmol/l and lower tHcy by about 1 μmol/l in populations selected based on high plasma tHcy concentrations (≥ 10 μmol/l, [68]) or not consuming vitamin supplements and RTEC [77]. Consumption of RTEC with 200 μg folate in combination with other vitamins did not result in different effects [77]. Homocysteine lowering effects were most effective in subjects with lowest plasma folate concentrations and highest baseline tHcy (tHcy reduction- 1.58 μmol/l and -1.87 μmol/l respectively) [77]. Venn et al [68] also tested fortification with 100 and 300 μg folate/portion and found similar tHcy results, concluding that 100 μg folate would be sufficient in population with ≥ 10 μmol /l plasma tHcy. Consumption of RTEC enriched with 440 μg folate in combination with RDA amounts of vitamin B 6 and B 12/portion resulted in small differences in plasma folate (7,5 nmol/l) and homocysteine (-0.4 μmol/l) in a population with in general already relatively high baseline folate and low homocysteine concentrations [66]. In addition, the reduction of the percentage of persons with high homocysteine (>10.4 μmol/l for women or 11.4 μmol/l for men) was greater in the supplemented group (13% to 3.2%) compared to the control group (10.4% to 7.3%). All three RCTs were funded by industry and showed a positive effect of the intervention.

#### Associations of RTEC consumption with BMI/weight gain

One prospective study in adults demonstrated that men consuming at least one portion of RTEC/day gained on average 0.59 and 0.46 kg less body weight after 8 and 13 years respectively than men consuming RTEC rarely [8] (Table 2). They also had a decreased risk of 22% and 12% to become overweight during 8 and 13 years of follow-up. Associations were also examined for whole grain and refined grain RTEC intake separately but these were not different.

Two prospective studies investigated the relationship between consumption of RTEC and BMI in children with a follow up of 7.5 [54] and 3 years [51] (Table 2). Both studies were secondary analyses of RCTs with 8–10 year old children including either both intervention groups [54] or the control group only [51]. Lower BMI was associated with more frequent RTEC consumption in both sexes in low-income minority children in one study [51] (every day of RTEC consumption decreased BMI by 2 percentiles), but only in boys in the other (BMI 20.4 vs 20.1, 0–3 times RTEC/week respectively) [54].

Negative associations of frequent RTEC consumption with body weight gain were found in all three RCTs, two of which [8;54] were funded by food industry.

#### Effects of RTEC consumption on body weight, satiety and food intake

Two RCTs, one in children (overweight or at risk of overweight [62]) and one in adults [11] investigated whether increase of consumption of RTEC results in a reduction in body weight (Table 3). Twelve weeks of RTEC consumption (≈ 33 g, different corn and rice based types) in combination with nutritional education not only prevented the weight gain observed in the other groups but it decreased weight (mean -1.01 kg) and body fat gain (0.8%) [62]. As these changes were different to that observed after the RTEC intervention without nutritional education, it can be assumed that nutritional education is responsible for this positive effect.

In adults, six weeks of consumption of RTEC (60 g for women/80 g for men) mainly in the morning instead of the habitual Finnish breakfast did not result in change in body weight (secondary objective) [11].

Both RCTs were funded by food industry and showed no effect of RTEC consumption (alone) on body weight reduction.

Seven RCTs [12;57;59;61;65;73;76] examined the effect of low DF vs high DF RTEC and/or wholemeal RTEC [65] on postprandial satiety and five of them also on subsequent energy intake [12;57;59;61;73] (Table 3). The amount of DF administered with the high DF RTEC varied between 2.3 g and 33 g whereas the control RTEC contained 0–4 g. The types of DF were wheat bran [12;61;65;73], b-glucan [59;76] and 2 types of arabinoxylans (AX): hydrolysed wheat bran AX and unhydrolysed flax AX [57]. Visual analog scales were applied in most studies to measure satiety and/or appetite and a questionnaire in one trial [73]. Three trials reported a significant difference in satiety/appetite measures. The degree of hunger was lower after ingestion of high versus low DF RTEC [73]. Furthermore, the average appetite score was highest after the high bran RTEC [12] and the ß-glucan RTECs resulted in a lower combined appetite score independent from dose [59].

Positive effects on satiety/appetite measured were found in two industrial funded [12; 59] and one not industrial funded RCT [73]. No effects were found in three industrial funded RCTs [57; 61;76] and one RCT without industrial funding [65].

In two trials [12;61] it was found that a large portion of RTEC (71 and 60 g) containing 33 and 28 g of mainly insoluble wheat fiber can reduce subsequent energy intake (Table 3). After breakfasts providing the same energy, food intake at an early subsequent meal (75 min) was reduced by 160 kcal [12]. In the other trial the lower caloric value of the high DF RTEC was not compensated at lunch (3 h later) resulting in lower cumulative energy intake (93 kcal) [61]. In another trial [73] two experiments were conducted with RTEC containing different amounts of wheat bran. In the first experiment a significant difference in cumulative (breakfast and lunch 3.5 h later) energy intake (≈ 140 kcal) was found after the RTECs with the highest (22 and 20 g) compared to that with the lowest DF content (0 g). In the second experiment comparing the RTEC with the lowest and highest amount of DF, a decrease of energy intake at lunch and of cumulative energy intake was observed after the high DF RTEC (≈ 100 and 200 kcal respectively). In a trial with overweight women consumption of RTEC enriched with 15 g AX (19 g total DF) did not result in decreased energy intake at lunch (4 h later) nor decreased cumulative energy intake compared to the low DF RTECs (4 and 3 g DF) [57]. In addition, RTEC enriched with a low amount of β-glucan (2.3–5.9 g) did not result in lower energy intake at lunch (4 h later) in overweight persons [59].

Positive effects of consumption of fiber-rich RTEC on subsequent food intake were found in two RCTs [12;61] funded by food industry and one RCT without industry-related funding [73], whereas the other two funded RCTs [57;59] showed no effect. One RCT (industry funded) investigated the effect of modifying the processing procedure of wheat flakes (sour-dough prefermentation, steam cooking omission, reduction sucrose content) on satiety. Modified wheat flakes successfully reduced hunger feelings at 120, 150 and 180 min after ingestion compared to conventionally produced wheat flakes and white wheat bread [60] (Table 3). The test meals had similar energy content and differed slightly in macronutrient and DF composition.

#### Associations of RTEC consumption with development of type 2 diabetes

One prospective study investigated the association between RTEC (cold breakfast cereals) consumption and incident diabetes in male physicians [55] (Table 2). Decreased risk of diabetes was clearly demonstrated for participants with a high consumption of whole grain RTEC (HR: 0.76 (95% CI 0.66–0.87) for 2–6 servings/wk, HR: 0.60 (95% CI 0.50–0.71) for ≥ 7 servings/wk), whereas the associations with consumption of refined RTEC were not significant in all groups (HR: 0.69 (95% CI 0.53–0.90) for 2–6 servings/wk, HR: 0.95 (95% CI 0.73–1.3) for ≥ 7 servings/wk) [55].

Another prospective study investigated the association between consumption of whole grain foods and incident diabetes in women [50] (Table 2). Analyses of HR of specific whole grain foods showed decrease risk of diabetes for high consumption of whole grain breakfast cereals ((HR: 0.71 (95% CI 0.62–0.82) for 5–6 servings/week and HR: 0.66 (95% CI 0.55–0.80) for ≥ 1/day).

Both these prospective studies showing associations between high consumption of whole grain RTEC and decreased risk of diabetes were not industry funded.

#### Effects of RTEC consumption on risk factors for type 2 diabetes

Two RCTs examined to what extent postprandial insulinemia is changed in response to RTEC with different content of DF and different GI [64;74] (Table 3). 136 g whole grain wheat RTEC enriched with corn bran (GI: 49, 50 g available carbohydrates, 63.5 g DF) compared to 60 g low DF RTEC (GI: 125, 50 g available carbohydrates, 2 g DF) consumed with water reduced postprandial the 2h-AUC of insulin by 50% in healthy volunteers [64]. Half the portion of those RTEC was administered with milk in the other trial in which 2h-AUC of insulin was only decreased (by 14%) in volunteers with high fasting insulin but not in those with normal insulin values [74]. Both those industrial funded RCTs found positive effects of fiber-rich RTEC on postprandial insulinemia.

Two RCTs investigated whether addition of soluble fiber to RTEC with the same carbohydrate content results in decreased postprandial glucose and insulin responses [59;70] (Table 3). In overweight volunteers, corn based RTEC with 4–6 g oat β-glucan did not reduce blood glucose but only decreased the 2h-AUC insulin by 14–17% compared to RTEC without β-glucan [59]. In healthy volunteers, however, addition of 4.5 g soluble fiber from guar gum to wheat RTEC decreased both the 2 h- AUC glucose and insulin by 47% and 34% respectively compared to control [70]. The industry funded RCT [59] found mixed results of consumption of RTEC rich in soluble fiber on postprandial glucose and insulin, whereas the RCT without industry-related funding [70] found reduction of both parameters.

One RCT (food-industry funded) investigated the effect of modified processing of wheat flakes (sourdough pre-fermentation, suppressing steam cooking) and reduced sucrose content on GI and insulinemic index (reference food was white wheat bread) [60] (Table 3). The GI of modified whole wheat flakes and standard whole wheat flakes was not different. However, the 90 min and 180 min insulinemic index of the modified flakes was decreased by 20 and 12% respectively.

Another RCT (food-industry funded) investigated whether the low GI of a DF-rich RTEC is caused by a slower rate of appearance of starch-derived glucose (Ra_gluc_, reflecting starch digestion) or a higher glucose uptake from the blood by tissues (Rd_gluc_) [67] (Table 3). The Ra_gluc_ of the high GI RTEC and low GI RTEC was not different. However, the Rd_gluc_ at 30–60 min was 31% higher after the low GI RTEC which was associated with a 125% higher 0–30 min insulin response. It was hypothesized that the higher protein content of the low GI RTEC (11 g) contributed to the higher insulin response and thereby increased Rd_gluc_ which could explain the low GI despite the same rate of starch digestion.

#### Effect of RTEC consumption on the composition of the colonic microbiota and on bowel function

One RCT (without industry-related funding) investigated the effect of 4-wk consumption of inulin-rich (9 g inulin/day) compared to inulin-free RTEC on the composition of the microbiota using selective growth media [69] (Table 3). It was found that the amount of bifidobacteria was higher after the inulin-rich RTEC compared to control, but only after correction for total anaerobes.

Two RCTs (food-industry funded) investigated the effect of 3-wk consumption of one portion whole grain RTEC/day on the composition of the microbiota with fluorescence in situ hybridization [10;58] (Table 3). One trial compared whole wheat RTEC (48 g, 5.7 g DF) to a wheat- bran based RTEC (48 g, 13 g DF) [10], whereas the other compared whole grain maize RTEC (48 g, 7 g DF) to refined maize RTEC (48 g, 0.4 g DF) [58]. In both trials, only whole grain RTEC consumption increased the amount of *Bifidobacterium* spp. compared to baseline. The increase in *Bifidobacterium* spp compared to control, however, was only significantly different in the wheat RTEC trial [10]. In this trial, also the numbers of *Lactobacillus/Enterococcus* were higher after the intervention with whole wheat RTEC compared to that with the wheat bran based RTEC.

The same three trials, which assessed the effect of DF-rich RTEC on the composition of the microbiota [10;58;69] (Table 3), monitored also bowel function as secondary outcome. A daily increase of DF in form of 9 g inulin or 7 g maize fiber did not change bowel habits [58;69]. During the intervention with wheat-bran based RTEC stool frequency was increased compared to that with whole wheat RTEC, and frequency of soft stools and flatulence increased [10]. Consumption of whole wheat RTEC resulted in more formed stool [10]. Consumption of DF-rich RTEC did not have an effect on bowel habits in one industrial funded RCT [58] and one without industry-related funding [69]. Another industrial funded RCT [10] reported improved bowel habits.

#### Association between RTEC consumption and cognitive decline

One prospective study (food-industry funded) investigated the association between frequency of RTEC consumption and cognitive decline in elderly subjects over 11 years [53] (Table 2). Daily consumers of RTEC had a pattern of cognitive decline similar to infrequent consumers.

#### Effect of RTEC consumption on acute cognitive performance

One RCT in children [75] and one in adolescents [63] investigated the effect of RTEC with low and high GI on acute cognitive performance (Table 3). The low GI RTEC (GI 30 [63] and 42 [75]) provided a lower amount of energy and carbohydrates, but higher amounts of protein than the high GI RTEC (both GI 77). In children, after the low GI RTEC secondary memory performance was better and decline in accuracy of attention was attenuated. Speed of attention and memory as well as working memory was not affected by GI [75]. In adolescents verbal episodic memory tasks were performed under divided attention which measured immediate, short-delay and long-delay memory [63]. No differences were found comparing the raw data scored after high and low GI RTEC consumption. However, when calculating remembering/ forgetting indices for each participant, it was shown that high GI RTEC improved long-delayed memory. Both RCTs were without industry-related funding and showed either positive [75] or negative effects [63] of consumption of low GI RTEC on specific cognitive tasks.

## Discussion

### Nutritional benefits

Frequent consumption (≥ 5 servings/week) of RTEC compared to low or no RTEC consumption consistently has been associated with a healthier dietary pattern in children and adults in most studies demonstrating a higher consumption of carbohydrates, DF and a reduction of total fat intake and cholesterol (only for children). Thus, current dietary recommendations are more likely to be met by RTEC consumers.

As many RTEC are fortified with micronutrients, it is not surprising that intake of those micronutrients is increased in RTEC consumers. However, increased micronutrient consumption is only relevant in case that micronutrient intake is below the nutritional recommendations. For this reason, we assessed the impact of RTEC consumption on micronutrient inadequacy. Our results show that the reduction of prevalence of inadequacy associated with frequent RTEC consumption is greatest for vitamin A, calcium, folate, vitamin B 6, magnesium and zinc. These results are mainly derived from surveys conducted in the US, Canada and Australia.

These data demonstrate that RTEC, due to fortification, DF content and by stimulating milk intake, can play an important role in reducing the prevalence of micronutrient inadequacy.

Of concern is the total sugar intake which was positively associated in children and adults with frequent RTEC consumption in most studies. Higher consumption of total sugar, which is the sum of free sugars, intrinsic sugars and milk sugars, can be partly explained by higher lactose intake due to an increase in milk consumption. However, it can also partly be due to the sugar content of RTEC (defined as “free” sugar) and dietary recommendations are to decrease ‘free’ sugar intake to less than 10% of the total daily energy consumption [79;80]. The current intake in some European countries and the US exceeds 10 energy% especially in children [81–83].

Analysis of different RTEC of leading brands in the US market showed that the mean sugar content of 142 types of RTEC was 28.1 g/100 g in 2006 but decreased to 24.8 g/100 g (mean of 151 types) in 2011 [84]. Even though this is a move in the right direction 24.8 g/100 g is still high. According to the color-coded Traffic Light System for classifying nutrients in solid foods of the Department of Health UK products containing >22.5 g/100 g would be colored red, indicating that this is not a healthy choice [85]. From this study it cannot be derived whether reductions were predominantly made in RTEC marketed to children or those not marketed to children (generic). This is of interest because it was shown that RTEC for children contained more sugar than generic RTEC (36 g/100 vs 23 g/100 g respectively in the US [86]; 28.2 g/100 g vs 18.1 g/100 g respectively in Germany [87]). Interestingly, in a RCT it was shown that children consuming either low-sugar or high sugar cereals did not differ in how much they liked the cereal [79]. Even though children added sugar to the low-sugar cereal they consumed half the amount of the sugar children in the high-sugar cereal group consumed. They were also more likely to put fresh fruit on their cereal as compared to the children in the high-sugar cereal group. This indicates that low-sugar RTEC are accepted by children and that the benefit of enhanced micronutrient intake due to RTEC consumption does not necessarily need to be accompanied by high sugar intake.

### Health benefits

#### Risk factors for CVD

Prospective studies that examined associations of low and high consumption of RTEC with health outcomes mostly differentiated between whole grain and refined grain RTEC. No studies were found that investigated associations of whole grain RTEC with cardiovascular disease directly. However, the associations with hypertension [52] and heart failure [56] were assessed and a decreased risk of 20 and 28% respectively was found. The inverse association of whole grain RTEC consumption with hypertension is consistent with that of a number of studies investigating associations with whole grain intake in general (women 0.89 [88], men 0.81 [89], young adults 0.83 HR [90]) whereas the magnitude of effect on heart failure was not comparable with that of a study examining the association with whole grain intake in general (0.93 HR [91]). Beneficial effects of whole grain products are related mainly to the bran fraction of the grain and its high content of micronutrients, like magnesium and zinc, and bioactive components, like phytic and ferulic acid, many having antioxidant properties [92;93]. Magnesium is one of the micronutrients linked to the prevention of hypertension [93;94] and oxidative stress is involved in the pathophysiology of cardiovascular disease and heart failure [95;96]. Furthermore, it is postulated that synergetic effects can occur as different components of whole grain act together to beneficial influence processes involved in development of disease [92].

In addition, hypocholesterolaemic properties of whole grain have been postulated that are mainly ascribed to viscous soluble fiber [97]. Reductions in total cholesterol and LDL were seen due to psyllium-enriched RTEC [71;72] but not with wheat bran RTEC in hypercholesterolemic men [71]. In normocholesterolemic persons RTEC based on whole grain maize [58], whole grain wheat and wheat bran [10] did not affect blood lipids. This is in agreement with the findings of a recent meta-analysis summarizing results of lipid-lowering effects of whole-grain interventions in apparently healthy [98]. Whole grain products based on wheat did not consistently exert lipid lowering effects in contrary to products based on barley and oat [48]. Psyllium fiber, like fiber in oat and barley, are soluble whereas wheat or corn fiber are mainly insoluble, which can explain these results.

Elevated plasma concentrations of homocysteine are suggested to be an additional risk factor for the development of cardiovascular disease [99], although not consistently [100]. Higher plasma folate concentrations are implicated with lower homocysteine [101] as well as a reduced risk of developing CVD [102]. Three studies consistently demonstrated that consumption of RTEC fortified with folate could increase plasma folate concentration and lower plasma homocysteine [66;68;77]. The effects were most pronounced in persons with low plasma folate and high homocysteine concentrations.

In summary, studies investigating whether RTEC consumption can reduce the risk of development of CVD addressed different risk factors. Prospective studies suggest that consumption of whole grain RTEC may reduce the risk of hypertension and heart failure, which so far has not been assessed in RCTs. RCTs demonstrated that RTEC with soluble fiber from psyllium have lipid lowering potency and folate-enriched RTEC can reduce plasma homocysteine concentrations. These prospective studies did not have industrial funding, whereas the effect of psyllium and folate-enriched RTEC were only investigated in RCTs which were industrial funded.

#### Weight gain/BMI, satiety and food intake

Lower weight gain (0.59 and 0.46 kg during 8 and 13 y respectively) and a lower risk of becoming obese (22 and 12%) was associated with frequent RTEC consumption in men without being different between refined and whole grain RTEC [8]. The magnitude of effect was similar in two prospective studies that examined the association between whole grain food and refined grain food intake [103;104]. Consumption of whole grain food resulted in 0.49 kg less weight gain during 8 y in men [103] and 0.39 kg less weight gain during 12 y in women [104]. In contrary to the RTEC study [8] refined grain intake was associated with an increase in weight in women (0.43 kg during 12 y) [104]. However, differences in weight gain found in these studies are quite small and its health impact is difficult to judge.

In 8–10 y old children frequency of RTEC consumption was associated with slightly lower BMI in boys in two prospective studies [51;54] but in girls only in one study (low-income minority children) [51]. These studies did not differentiate between types of RTEC. Similar associations between BMI and consumption of breakfast cereals in general (9–10 y girls [105]) or whole grain foods (13–15 y old boys and girls [106]) were found, with no sex-related differences. Explanations for these beneficial effects postulated are the more healthy eating pattern of RTEC consumers with increased intakes of whole grain, DF and reduced fat or increased satiety [51;54] and higher insulin sensitivity in case of whole grain consumers [106]. Results of two RCTs [11;62] investigating the effect of high vs low RTEC consumption on body weight do not substantiate results from prospective studies. However, in both trials RTEC with a low content of DF were administered and one was of relative short duration.

Other RCTs explored the effect of DF-rich RTEC on postprandial satiety and food intake. From these trials it seems that postprandial satiety and/or appetite is not affected by higher DF content of RTEC, as only three from seven studies reported a decrease [12;59;73]. However, consumption of wheat bran RTEC decreased energy intake at a subsequent meal in normal weight subjects [12;61;73].

In summary, consumption of RTEC (all types) is associated with modest reduction in weight gain or BMI in adults and children in prospective studies, which so far is not substantiated with RCTs. There are indications that RTEC enriched with wheat bran can decrease energy intake at a subsequent meal in normal weight persons, with RCTs without industry-related funding showing similar results as industrial funded RCTs. However, long term RCTs are needed to demonstrate that this results in decreased weight gain. Furthermore, it seems of great interest to not only assess body weight but also fat mass as the results of a recent meta-analyses demonstrated that whole grain interventions can decrease fat mass, despite no effect on body weight [107].

#### Type 2 diabetes and risk factors

Consumption of whole grain RTEC 2–6 times weekly was associated with a decreased the risk of the development of type 2 diabetes by 24% and 29% and ≥ 7 servings/week by 40 and 43% [55;50]. These finding are consistent with that of studies investigating associations with total whole grain intake (0.79 HR [108], 0.67 [109], 0.72 [110]). Increased intake of bran-derived micronutrients like magnesium and zinc as well as bioactive components may contribute to beneficial effects [92;93]. Magnesium for example, plays an important role in insulin sensitivity [94;111;112] and recently the property of zinc to influence synthesis, secretion and the action of insulin has become clear [113]. In addition, chronic low-grade inflammation and oxidative stress are factors involved in the development of type 2 diabetes, which can be alleviated by certain micronutrients as well as by bioactive compounds,—possibly through synergistic action [92].

Reduction of postprandial glucose and/or insulin concentrations are considered beneficial as repeated high glucose concentrations and related high insulin concentrations can lead to decreased insulin sensitivity and β-cell function in susceptible persons [114;115]. Reductions of postprandial glucose and insulin concentrations have been demonstrated for viscous soluble fiber [116]. The property of RTEC enriched with soluble fiber to decrease postprandial glucose and insulin was shown for guar gum (4.5 g) in healthy [70], whereas in overweight persons only the insulin response was reduced after RTEC with oat b-glucan [59].

Evidence from prospective studies, which were all without food-industry related funding, indicates a reduced risk of development of type 2 diabetes due to consumption of whole grain RTEC. However, RCTs investigating the effect of whole grain versus refined grain RTEC on risk factors related to the development of type 2 diabetes are needed to draw a definite conclusion. Addition of soluble fiber seems a promising strategy to reduce not only postprandial glucose but also insulin concentrations (independent from funding sources) and deserves further evaluation. Lower postprandial insulin response is considered beneficial because this would be less demanding for the pancreatic β-cells [115] and could play a role in preventing insulin resistance [117]. In addition, more recently, diets with a low insulin load were reported to be associated with lower body fat during puberty [118] and with lower energy intake in obese adolescents with features of insulin resistance and/or prediabetes [119].

### Strengths and Limitations

This review provides a comprehensive overview of nutritional and health effects that are related to the consumption of RTEC and has identified specific favorable characteristics. One of the review’s strength is the careful selection of studies, excluding studies with cooked cereals or those in which cereals were not defined. In addition, RCTs were not considered in which RTEC were administered at several occasions during the day or breakfasts included other products than RTEC, milk and fruit. This enables us to draw conclusion on the properties of RTEC only. Data about nutritional benefits were derived from large, national representative surveys conducted in a number of different countries in many age-groups, which aids generalizability of results. There are some limitations of this review and the body of evidence. Evidence for nutritional and health benefits is partly derived from observational studies in which dietary data are self-reported. These studies are more prone to bias and confounding than RCTs, therefore results have to be interpreted with caution. For assessing health benefits, however, only prospective studies and no cross-sectional studies, which lack temporal relationship, have been used. As prospective studies were mainly conducted in the US generalizability of these results is uncertain. A large number of studies (45) were (partly) funded by food-industry, which can introduce reporting bias. As we examined and discussed the results also in view of funding sources, we can conclude that reporting bias seems less likely what concerns the prospective studies and most RCTs.

## Conclusion

Frequent consumption of RTEC (≥ 5 servings/week) as compared to no or low RTEC consumption is associated with a healthier dietary pattern, concerning intake of carbohydrates, DF, fat and micronutrients, however total sugar intake is higher. The impact of frequent RTEC consumption on inadequacy of micronutrient intake is highest for vitamin A, calcium, folate, vitamin B 6, magnesium and zinc.

Evidence from prospective studies suggests that whole grain RTEC may have beneficial effects on hypertension and type 2 diabetes. These protective effects seem biological plausible, however, to prove a causal relationship RCTs are needed that assess the effect of whole grain versus refined grain RTEC on hypertension and risk factors for type 2 diabetes.

Consumption of RTEC with soluble fiber from psyllium helps to reduce LDL and total cholesterol in hypercholesterolemic men. RTEC fortified with folate have the potency to reduce plasma homocysteine especially in persons with low folate and high homocysteine plasma concentrations. Addition of soluble fiber to RTEC could aid in reducing postprandial glycaemia and insulinemia but more studies are needed to draw a final conclusion. The effect of RTEC on body weight, intestinal health and cognitive function needs further evaluation.

## Supporting Information

S1 Checklist PRISMA checklist for the Reporting of Systematic Reviews of Randomized controlled trials. (DOC)

S2 Checklist MOOSE checklist for the Reporting of Meta-analyses of Observational Studies. (DOCX)

S1 Protocol (DOC)

S1 Table Differences in daily intake of energy, macronutrients, cholesterol, dietary fiber and sodium of frequent versus low/no RTEC consumers. (DOCX)

S2 Table Percentage of population with daily intake of micronutrients below recommended intake by frequency of RTEC consumption. (DOCX)

## References

[1] Rampersaud GC, Pereira MA, Girard BL, Adams J, Metzl JD. Breakfast habits, nutritional status, body weight, and academic performance in children and adolescents. J Am Diet Assoc. 2005 5;105(5):743–60. 10.1016/j.jada.2005.02.007 15883552

[2] Szajewska H, Ruszczynski M. Systematic review demonstrating that breakfast consumption influences body weight outcomes in children and adolescents in Europe. Crit Rev Food Sci Nutr. 2010 2;50(2):113–9. 10.1080/10408390903467514 20112153

[3] Hoyland A, Dye L, Lawton CL. A systematic review of the effect of breakfast on the cognitive performance of children and adolescents. Nutr Res Rev. 2009 12;22(2):220–43. 10.1017/S0954422409990175 19930787

[4] Bi H, Gan Y, Yang C, Chen Y, Tong X, Lu Z. Breakfast skipping and the risk of type 2 diabetes: a meta-analysis of observational studies. Public Health Nutr. 2015 11;18(16):3013–9. 10.1017/S1368980015000257 25686619 PMC10271832

[5] Cahill LE, Chiuve SE, Mekary RA, Jensen MK, Flint AJ, Hu FB, et al Prospective study of breakfast eating and incident coronary heart disease in a cohort of male US health professionals. Circulation. 2013 7 23;128(4):337–43. 10.1161/CIRCULATIONAHA.113.001474 23877060 PMC3797523

[6] Smith KJ, Gall SL, McNaughton SA, Blizzard L, Dwyer T, Venn AJ. Skipping breakfast: longitudinal associations with cardiometabolic risk factors in the Childhood Determinants of Adult Health Study. Am J Clin Nutr. 2010 12;92(6):1316–25. 10.3945/ajcn.2010.30101 20926520

[7] Albertson AM, Tobelmann RC. Impact of Ready-To-Eat Cereal Consumption on the Diets of Children 7–12 Years old. Cereal Foods World. 1993;38:428–31.

[8] Bazzano LA, Song Y, Bubes V, Good CK, Manson JE, Liu S. Dietary intake of whole and refined grain breakfast cereals and weight gain in men. Obes Res. 2005 11;13(11):1952–60. 10.1038/oby.2005.240 16339127

[9] Bertrais S, Polo Luque ML, Preziosi P, Fieux B, Torra De FM, Galan P, et al Contribution of ready-to-eat cereals to nutrition intakes in French adults and relations with corpulence. Ann Nutr Metab. 2000;44(5–6):249–55. 11146332 10.1159/000046692

[10] Costabile A, Klinder A, Fava F, Napolitano A, Fogliano V, Leonard C, et al Whole-grain wheat breakfast cereal has a prebiotic effect on the human gut microbiota: a double-blind, placebo-controlled, crossover study. Br J Nutr. 2008 1;99(1):110–20. 10.1017/S0007114507793923 17761020

[11] Kleemola P, Puska P, Vartiainen E, Roos E, Luoto R, Ehnholm C. The effect of breakfast cereal on diet and serum cholesterol: a randomized trial in North Karelia, Finland. Eur J Clin Nutr. 1999 9;53(9):716–21. 10509768 10.1038/sj.ejcn.1600849

[12] Samra RA, Anderson GH. Insoluble cereal fiber reduces appetite and short-term food intake and glycemic response to food consumed 75 min later by healthy men. Am J Clin Nutr. 2007 10;86(4):972–9. 17921373 10.1093/ajcn/86.4.972

[13] de la Hunty A, Gibson S, Ashwell M. Does regular breakfast cereal consumption help children and adolescents stay slimmer? A systematic review and meta-analysis. Obes Facts. 2013;6(1):70–85. 10.1159/000348878 23466487 PMC5644749

[14] Timlin MT, Pereira MA. Breakfast frequency and quality in the etiology of adult obesity and chronic diseases. Nutr Rev. 2007 6;65(6 Pt 1):268–81. 17605303 10.1301/nr.2007.jun.268-281

[15] Williams PG. The benefits of breakfast cereal consumption: a systematic review of the evidence base. Adv Nutr. 2014 9;5(5):636S–73S. 10.3945/an.114.006247 25225349 PMC4188247

[16] Moher D, Liberati A, Tetzlaff J, Altman DG. Preferred reporting items for systematic reviews and meta-analyses: the PRISMA statement. PLoS Med. 2009 7 21;6(7):e1000097 10.1371/journal.pmed.1000097 19621072 PMC2707599

[17] Stroup DF, Berlin JA, Morton SC, Olkin I, Williamson GD, Rennie D, et al Meta-analysis of observational studies in epidemiology: a proposal for reporting. Meta-analysis Of Observational Studies in Epidemiology (MOOSE) group. JAMA. 2000 4 19;283(15):2008–12. 10789670 10.1001/jama.283.15.2008

[18] Priebe MG, van Binsbergen JJ, de VR, Vonk RJ. Whole grain foods for the prevention of type 2 diabetes mellitus. Cochrane Database Syst Rev. 2008;(1):CD006061 10.1002/14651858.CD006061.pub2 18254091 PMC10384032

[19] Higgins JP, Altman DG, Gotzsche PC, Juni P, Moher D, Oxman AD, et al The Cochrane Collaboration's tool for assessing risk of bias in randomised trials. BMJ. 2011;343:d5928 10.1136/bmj.d5928 22008217 PMC3196245

[20] Papoutsou S, Briassoulis G, Hadjigeorgiou C, Savva SC, Solea T, Hebestreit A, et al The combination of daily breakfast consumption and optimal breakfast choices in childhood is an important public health message. Int J Food Sci Nutr. 2014 2 11. 10.3109/09637486.2013.854750 24512299

[21] Koo HC, Abdul Jalil SN, Ruzita AT. Breakfast Eating Pattern and Ready-to-Eat Cereals Consumption among Schoolchildren in Kuala Lumpur. Malays J Med Sci. 2015 1;22(1):32–9. 25892948 PMC4390772

[22] Barr SI, DiFrancesco L, Fulgoni VL III. Breakfast consumption is positively associated with nutrient adequacy in Canadian children and adolescents. Br J Nutr. 2014 10 28;112(8):1373–83. 10.1017/S0007114514002190 25196844 PMC4197762

[23] Affenito SG, Thompson D, Dorazio A, Albertson AM, Loew A, Holschuh NM. Ready-to-eat cereal consumption and the School Breakfast Program: relationship to nutrient intake and weight. J Sch Health. 2013 1;83(1):28–35. 10.1111/j.1746-1561.2012.00744.x 23253288

[24] Barr SI, DiFrancesco L, Fulgoni VL III. Consumption of breakfast and the type of breakfast consumed are positively associated with nutrient intakes and adequacy of Canadian adults. J Nutr. 2013 1;143(1):86–92. 10.3945/jn.112.167098 23173176

[25] Albertson AM, Wold AC, Joshi N. Ready-to-Eat Cereal Consumption Patterns: The Relationship to Nutrient Intake, Whole Grain Intake, and Body Mass Index in an Older American Population. J Aging Res. 2012;2012:631310 10.1155/2012/631310 23094158 PMC3474243

[26] Grieger JA, Cobiac L. Comparison of dietary intakes according to breakfast choice in Australian boys. Eur J Clin Nutr. 2012 6;66(6):667–72. 10.1038/ejcn.2011.220 22234045

[27] Albertson AM, Thompson DR, Franko DL, Holschuh NM. Weight indicators and nutrient intake in children and adolescents do not vary by sugar content in ready-to-eat cereal: results from National Health and Nutrition Examination Survey 2001–2006. Nutr Res. 2011 3;31(3):229–36. 10.1016/j.nutres.2011.03.004 21481717

[28] Deshmukh-Taskar PR, Radcliffe JD, Liu Y, Nicklas TA. Do breakfast skipping and breakfast type affect energy intake, nutrient intake, nutrient adequacy, and diet quality in young adults? NHANES 1999–2002. J Am Coll Nutr. 2010 8;29(4):407–18. 21041816 10.1080/07315724.2010.10719858

[29] Deshmukh-Taskar PR, Nicklas TA, O'Neil CE, Keast DR, Radcliffe JD, Cho S. The relationship of breakfast skipping and type of breakfast consumption with nutrient intake and weight status in children and adolescents: the National Health and Nutrition Examination Survey 1999–2006. J Am Diet Assoc. 2010 6;110(6):869–78. 10.1016/j.jada.2010.03.023 20497776

[30] Montenegro-Bethancourt G, Vossenaar M, Kuijper LD, Doak CM, Solomons NW. Ready-to-eat cereals are key sources of selected micronutrients among schoolchildren from public and private elementary schools in Quetzaltenango, Guatemala. Nutr Res. 2009 5;29(5):335–42. 10.1016/j.nutres.2009.05.003 19555815

[31] Williams BM, O'Neil CE, Keast DR, Cho S, Nicklas TA. Are breakfast consumption patterns associated with weight status and nutrient adequacy in African-American children? Public Health Nutr. 2009 4;12(4):489–96. 10.1017/S1368980008002760 18503723

[32] Song WO, Chun OK, Kerver J, Cho S, Chung CE, Chung SJ. Ready-to-eat breakfast cereal consumption enhances milk and calcium intake in the US population. J Am Diet Assoc. 2006 11;106(11):1783–9. 10.1016/j.jada.2006.08.015 17081829

[33] van den Boom A, Serra-Majem L, Ribas L, Ngo J, Perez-Rodrigo C, Aranceta J, et al The contribution of ready-to-eat cereals to daily nutrient intake and breakfast quality in a Mediterranean setting. J Am Coll Nutr. 2006 4;25(2):135–43. 16582030 10.1080/07315724.2006.10719524

[34] Song WO, Chun OK, Obayashi S, Cho S, Chung CE. Is consumption of breakfast associated with body mass index in US adults? J Am Diet Assoc. 2005 9;105(9):1373–82. 10.1016/j.jada.2005.06.002 16129078

[35] Kafatos A, Linardakis M, Bertsias G, Mammas I, Fletcher R, Bervanaki F. Consumption of ready-to-eat cereals in relation to health and diet indicators among school adolescents in Crete, Greece. Ann Nutr Metab. 2005 5;49(3):165–72. 10.1159/000086880 16006785

[36] Albertson AM, Anderson GH, Crockett SJ, Goebel MT. Ready-to-eat cereal consumption: its relationship with BMI and nutrient intake of children aged 4 to 12 years. J Am Diet Assoc. 2003 12;103(12):1613–9. 10.1016/j.jada.2003.09.020 14647087

[37] Galvin MA, Kiely M, Flynn A. Impact of ready-to-eat breakfast cereal (RTEBC) consumption on adequacy of micronutrient intakes and compliance with dietary recommendations in Irish adults. Public Health Nutr. 2003 6;6(4):351–63. 10.1079/PHN2002441 12795823

[38] Preziosi P, Galan P, Deheeger M, Yacoub N, Drewnowski A, Hercberg S. Breakfast type, daily nutrient intakes and vitamin and mineral status of French children, adolescents, and adults. J Am Coll Nutr. 1999 4;18(2):171–8. 10204834 10.1080/07315724.1999.10718846

[39] McNulty H, Eaton-Evans J, Cran G, Woulahan G, Boreham C, Savage JM, et al Nutrient intakes and impact of fortified breakfast cereals in schoolchildren. Arch Dis Child. 1996 12;75(6):474–81. 9014598 10.1136/adc.75.6.474 PMC1511814

[40] Ruxton CH, O'Sullivan KR, Kirk TR, Belton NR, Holmes MA. The contribution of breakfast to the diets of a sample of 136 primary-schoolchildren in Edinburgh. Br J Nutr. 1996 3;75(3):419–31. 8785215 10.1079/bjn19960144

[41] Ortega RM, Requejo AM, Redondo R, Lopez-Sobaler AM, Andres P, Ortega A, et al Influence of the intake of fortified breakfast cereals on dietary habits and nutritional status of Spanish schoolchildren. Ann Nutr Metab. 1996;40(3):146–56. 8862697 10.1159/000177908

[42] Nicklas TA, Myers L, Berenson GS. Total nutrient intake and ready-to-eat cereal consumption of children and young adults in the Bogalusa Heart Study. Nutr Rev. 1995 9;53(9 Pt 2):S39–S45. 8577417

[43] Morgan KJ, Zabik ME, Leveille GA. The role of breakfast in nutrient intake of 5- to 12-year-old children. Am J Clin Nutr. 1981 7;34(7):1418–27. 6266245 10.1093/ajcn/34.7.1418

[44] Albertson AM, Franko DL, Thompson DR, Tuttle C, Holschuh NM. Ready-to-Eat Cereal Intake is Associated with an Improved Nutrient Intake Profile among Food Insecure Children in the United States. J Hunger Environ Nutr. 2013;8(2):200–20.

[45] Grieger J, Kim S, Cobiac L. Where do Australian children get their dietary fibre? A focus on breakfast food choices. Nutrition & Dietetics. 2013 6;70(2):132–8.

[46] Albertson AM, Affenito SG, Culp JM, Buklis P, Joshi N. The Association Between Ready-to-Eat Cereal Consumption, Nutrient Intakes of the Canadian Population 12 Years and Older and Body Weight Measures: Results From a Nationally Representative Canadian Population. J Food Res. 2013;2(3):11–21.

[47] Sampson A, Dixit S, Meyers A, Houser R. The nutritional impact of breakfast consumption on the diets of inner-city African-American elementary school children. J Natl Med Assoc. 1995;87:195–202. 7731069 PMC2607827

[48] Morgan KJ, Zabik ME, Stampley GL. Breakfast consumption patterns of U.S. children and adolescents. Nutr Res. 1986;6:635–46.

[49] Yeung LF, Cogswell ME, Carriquiry AL, Bailey LB, Pfeiffer CM, Berry RJ. Contributions of enriched cereal-grain products, ready-to-eat cereals, and supplements to folic acid and vitamin B-12 usual intake and folate and vitamin B-12 status in US children: National Health and Nutrition Examination Survey (NHANES), 2003–2006. Am J Clin Nutr. 2011 1;93(1):172–85. 10.3945/ajcn.2010.30127 21084645

[50] Liu S, Manson JE, Stampfer MJ, Hu FB, Giovannucci E, Colditz GA, et al A prospective study of whole-grain intake and risk of type 2 diabetes mellitus in US women. Am J Public Health. 2000 9;90(9):1409–15. 10983198 10.2105/ajph.90.9.1409 PMC1447620

[51] Balvin FL, Trevino RP, Echon RM, Garcia-Dominic O, DiMarco N. Association between frequency of ready-to-eat cereal consumption, nutrient intakes, and body mass index in fourth- to sixth-grade low-income minority children. J Acad Nutr Diet. 2013 4;113(4):511–9. 23465566 10.1016/j.jand.2013.01.006

[52] Kochar J, Gaziano JM, Djousse L. Breakfast cereals and risk of hypertension in the Physicians' Health Study I. Clin Nutr. 2012 2;31(1):89–92. 10.1016/j.clnu.2011.08.001 21868140 PMC3289098

[53] Wengreen H, Nelson C, Munger RG, Corcoran C. Prospective study of ready-to-eat breakfast cereal consumption and cognitive decline among elderly men and women. J Nutr Health Aging. 2011 3;15(3):202–7. 21369668 10.1007/s12603-010-0303-7 PMC4533994

[54] Albertson AM, Affenito SG, Bauserman R, Holschuh NM, Eldridge AL, Barton BA. The relationship of ready-to-eat cereal consumption to nutrient intake, blood lipids, and body mass index of children as they age through adolescence. J Am Diet Assoc. 2009 9;109(9):1557–65. 10.1016/j.jada.2009.06.363 19699835

[55] Kochar J, Djousse L, Gaziano JM. Breakfast cereals and risk of type 2 diabetes in the Physicians' Health Study I. Obesity (Silver Spring). 2007 12;15(12):3039–44. 18198313 10.1038/oby.2007.362

[56] Djousse L, Gaziano JM. Breakfast cereals and risk of heart failure in the physicians' health study I. Arch Intern Med 2007 10 22;167(19):2080–5. 10.1001/archinte.167.19.2080 17954802

[57] Lafond DW, Greaves KA, Maki KC, Leidy HJ, Romsos DR. Effects of two dietary fibers as part of ready-to-eat cereal (RTEC) breakfasts on perceived appetite and gut hormones in overweight women. Nutrients. 2015;7(2):1245–66. 10.3390/nu7021245 25689743 PMC4344586

[58] Carvalho-Wells AL, Helmolz K, Nodet C, Molzer C, Leonard C, McKevith B, et al Determination of the in vivo prebiotic potential of a maize-based whole grain breakfast cereal: a human feeding study. Br J Nutr. 2010 11;104(9):1353–6. 10.1017/S0007114510002084 20487589

[59] Beck EJ, Tosh SM, Batterham MJ, Tapsell LC, Huang XF. Oat beta-glucan increases postprandial cholecystokinin levels, decreases insulin response and extends subjective satiety in overweight subjects. Mol Nutr Food Res. 2009 10;53(10):1343–51. 10.1002/mnfr.200800343 19753601

[60] Lioger D, Fardet A, Foassert P, Davicco MJ, Mardon J, Gaillard-Martinie B, et al Influence of sourdough prefermentation, of steam cooking suppression and of decreased sucrose content during wheat flakes processing on the plasma glucose and insulin responses and satiety of healthy subjects. J Am Coll Nutr. 2009 2;28(1):30–6. 19571157 10.1080/07315724.2009.10719758

[61] Hamedani A, Akhavan T, Samra RA, Anderson GH. Reduced energy intake at breakfast is not compensated for at lunch if a high-insoluble-fiber cereal replaces a low-fiber cereal. Am J Clin Nutr. 2009 5;89(5):1343–9. 10.3945/ajcn.2008.26827 19339400

[62] Rosado JL, del RA, Montemayor K, Garcia OP, Caamano MC. An increase of cereal intake as an approach to weight reduction in children is effective only when accompanied by nutrition education: a randomized controlled trial. Nutr J. 2008;7:28 10.1186/1475-2891-7-28 18783622 PMC2543040

[63] Smith MA, Foster JK. The impact of a high versus a low glycaemic index breakfast cereal meal on verbal episodic memory in healthy adolescents. Nutr Neurosci. 2008 10;11(5):219–27. 10.1179/147683008X344110 18782482

[64] Barkoukis H, Marchetti CM, Nolan B, Sistrun SN, Krishnan RK, Kirwan JP. A high glycemic meal suppresses the postprandial leptin response in normal healthy adults. Ann Nutr Metab. 2007;51(6):512–8. 10.1159/000112309 18073462

[65] Hlebowicz J, Wickenberg J, Fahlstrom R, Bjorgell O, Almer LO, Darwiche G. Effect of commercial breakfast fibre cereals compared with corn flakes on postprandial blood glucose, gastric emptying and satiety in healthy subjects: a randomized blinded crossover trial. Nutr J. 2007;6:22 10.1186/1475-2891-6-22 17875200 PMC2031888

[66] Tucker KL, Olson B, Bakun P, Dallal GE, Selhub J, Rosenberg IH. Breakfast cereal fortified with folic acid, vitamin B-6, and vitamin B-12 increases vitamin concentrations and reduces homocysteine concentrations: a randomized trial. Am J Clin Nutr. 2004 5;79(5):805–11. 15113718 10.1093/ajcn/79.5.805

[67] Schenk S, Davidson CJ, Zderic TW, Byerley LO, Coyle EF. Different glycemic indexes of breakfast cereals are not due to glucose entry into blood but to glucose removal by tissue. Am J Clin Nutr. 2003 10;78(4):742–8. 14522732 10.1093/ajcn/78.4.742

[68] Venn BJ, Mann JI, Williams SM, Riddell LJ, Chisholm A, Harper MJ, et al Assessment of three levels of folic acid on serum folate and plasma homocysteine: a randomised placebo-controlled double-blind dietary intervention trial. Eur J Clin Nutr. 2002 8;56(8):748–54. 10.1038/sj.ejcn.1601388 12122551

[69] Brighenti F, Casiraghi MC, Canzi E, Ferrari A. Effect of consumption of a ready-to-eat breakfast cereal containing inulin on the intestinal milieu and blood lipids in healthy male volunteers. Eur J Clin Nutr. 1999 9;53(9):726–33. 10509770 10.1038/sj.ejcn.1600841

[70] Fairchild RM, Ellis PR, Byrne AJ, Luzio SD, Mir MA. A new breakfast cereal containing guar gum reduces postprandial plasma glucose and insulin concentrations in normal-weight human subjects. Br J Nutr. 1996 7;76(1):63–73. 8774217 10.1079/bjn19960009

[71] Roberts DC, Truswell AS, Bencke A, Dewar HM, Farmakalidis E. The cholesterol-lowering effect of a breakfast cereal containing psyllium fibre. Med J Aust. 1994 12 5;161(11–12):660–4. 7830631

[72] Bell LP, Hectorn KJ, Reynolds H, Hunninghake DB. Cholesterol-lowering effects of soluble-fiber cereals as part of a prudent diet for patients with mild to moderate hypercholesterolemia. Am J Clin Nutr. 1990 12;52(6):1020–6. 2173390 10.1093/ajcn/52.6.1020

[73] Levine AS, Tallman JR, Grace MK, Parker SA, Billington CJ, Levitt MD. Effect of breakfast cereals on short-term food intake. Am J Clin Nutr. 1989 12;50(6):1303–7. 2556910 10.1093/ajcn/50.6.1303

[74] Wolever TMS, Campbell JE, Geleva D, Anderson GH. High-fiber cereal reduces postprandial insulin responses in hyperinsulinemic but not normoinsulinemic subjects. Diabetes Care. 2004;27(6):1281–5. 15161776 10.2337/diacare.27.6.1281

[75] Ingwersen J, Defeyter MA, Kennedy DO, Wesnes KA, Scholey AB. A low glycaemic index breakfast cereal preferentially prevents children's cognitive performance from declining throughout the morning. Appetite. 2007;49(1):240–4. 10.1016/j.appet.2006.06.009 17224202

[76] Hlebowicz J, Darwiche G, Bjorgell O, Almer LO. Effect of muesli with 4 g oat (beta)-glucan on postprandial blood glucose, gastric emptying and satiety in healthy subjects: A randomized crossover trial. J Am Coll Nutr. 2008;27(4):470–5. 18978166 10.1080/07315724.2008.10719727

[77] Schorah CJ, Devitt H, Lucock M, Dowell AC. The responsiveness of plasma homocysteine to small increases in dietary folic acid: a primary care study. Eur J Clin Nutr. 1998 6;52(6):407–11. 9683392 10.1038/sj.ejcn.1600576

[78] Morgan KJ, Zabik ME, Stampley GL. The role of breakfast in diet adequacy of the U.S. adult population. J Am Coll Nutr. 1986;5(6):551–63. 3782651 10.1080/07315724.1986.10720156

[79] Harris JL, Schwartz MB, Ustjanauskas A, Ohri-Vachaspati P, Brownell KD. Effects of serving high-sugar cereals on children's breakfast-eating behavior. Pediatrics 2011 1;127(1):71–6. 10.1542/peds.2010-0864 21149436

[80] World Health Organisation. Guideline: Sugars intake for adults and chldren. 2015. 25905159

[81] EFSA Panel on Dietetic Products NaA. Scientific opinion on dietary reference values for carbohydrates and dietary fibre. EFSA Journal. 2010;8(3):1463–549.

[82] Ervin RB, Ogden C.L. Consumption of added sugars among U.S. aduots, 2005–2010 NCHS data brief, no 122 Hyattsville, MD: National Center for Health Statistics 2013. 23742909

[83] Ervin RB, Kit BK, Carroll MD, Ogden C.L. Consumption of added sugar among U.S. children and adolescents, 2005–2008 NCHS data brief, no 87 Hyattsville, MD: National Center for Health Statistics 2012.

[84] Thomas RD, Pehrsson PR, Ahuja JKC, Smieja E, Miller KB. Recent trends in ready-to-eat breakfast cereals in the U.S. Procedia Food Science. 2013;2:20–6.

[85] Department of Health UK. Guide to creating a front of pack (FoP) nutrition label for pre-packed products sold through retail outlets. June 2013. https://www.gov.uk/government/publications/front-of-pack-nutrition-labelling-guidance. 2016.

[86] Schwartz MB, Vartanian LR, Wharton CM, Brownell KD. Examining the nutritional quality of breakfast cereals marketed to children. J Am Diet Assoc. 2008 4;108(4):702–5. 10.1016/j.jada.2008.01.003 18375229

[87] Germer S, Hilzendegen C, Stroebele-Benschop N. Sugar content of German breakfast cereals for children—recommendations and reality. Ernaehrungs Umschau international. 2013;60(6):89–95.

[88] Wang L, Gaziano JM, Liu S, Manson JE, Buring JE, Sesso HD. Whole- and refined-grain intakes and the risk of hypertension in women. Am J Clin Nutr. 2007 8;86(2):472–9. 17684221 10.1093/ajcn/86.2.472

[89] Flint AJ, Hu FB, Glynn RJ, Jensen MK, Franz M, Sampson L, et al Whole grains and incident hypertension in men. Am J Clin Nutr. 2009 9;90(3):493–8. 10.3945/ajcn.2009.27460 19571218 PMC2728640

[90] Steffen LM, Kroenke CH, Yu X, Pereira MA, Slattery ML, Van HL, et al Associations of plant food, dairy product, and meat intakes with 15-y incidence of elevated blood pressure in young black and white adults: the Coronary Artery Risk Development in Young Adults (CARDIA) Study. Am J Clin Nutr. 2005 12;82(6):1169–77. 16332648 10.1093/ajcn/82.6.1169

[91] Nettleton JA, Steffen LM, Loehr LR, Rosamond WD, Folsom AR. Incident heart failure is associated with lower whole-grain intake and greater high-fat dairy and egg intake in the Atherosclerosis Risk in Communities (ARIC) study. J Am Diet Assoc. 2008 11;108(11):1881–7. 10.1016/j.jada.2008.08.015 18954578 PMC2650810

[92] Fardet A. New hypotheses for the health-protective mechanisms of whole-grain cereals: what is beyond fibre? Nutr Res Rev. 2010 6;23(1):65–134. 10.1017/S0954422410000041 20565994

[93] Lillioja S, Neal AL, Tapsell L, Jacobs DR Jr. Whole grains, type 2 diabetes, coronary heart disease, and hypertension: links to the aleurone preferred over indigestible fiber. Biofactors. 2013 5;39(3):242–58. 10.1002/biof.1077 23355358 PMC3640698

[94] Grober U, Schmidt J, Kisters K. Magnesium in Prevention and Therapy. Nutrients. 2015 9;7(9):8199–226. 10.3390/nu7095388 26404370 PMC4586582

[95] Donato AJ, Morgan RG, Walker AE, Lesniewski LA. Cellular and molecular biology of aging endothelial cells. J Mol Cell Cardiol. 2015 2 2. 10.1016/j.yjmcc.2015.01.021 PMC4522407 25655936

[96] Tsutsui H, Kinugawa S, Matsushima S. Oxidative stress and heart failure. Am J Physiol Heart Circ Physiol. 2011 12;301(6):H2181–H2190. 10.1152/ajpheart.00554.2011 21949114

[97] Truswell AS. Cereal grains and coronary heart disease. Eur J Clin Nutr. 2002 1;56(1):1–14. 10.1038/sj.ejcn.1601283 11840174

[98] Hollaender PL, Ross AB, Kristensen M. Whole-grain and blood lipid changes in apparently healthy adults: a systematic review and meta-analysis of randomized controlled studies. Am J Clin Nutr. 2015 9;102(3):556–72. 10.3945/ajcn.115.109165 26269373

[99] Ueland PM, Refsum H, Beresford SA, Vollset SE. The controversy over homocysteine and cardiovascular risk. Am J Clin Nutr. 2000 8;72(2):324–32. 10919921 10.1093/ajcn/72.2.324

[100] Brattstrom L, Wilcken DE. Homocysteine and cardiovascular disease: cause or effect? Am J Clin Nutr. 2000 8;72(2):315–23. 10919920 10.1093/ajcn/72.2.315

[101] Selhub J, Jacques PF, Rosenberg IH, Rogers G, Bowman BA, Gunter EW, et al Serum total homocysteine concentrations in the third National Health and Nutrition Examination Survey (1991–1994): population reference ranges and contribution of vitamin status to high serum concentrations. Ann Intern Med. 1999 9 7;131(5):331–9. 10475885 10.7326/0003-4819-131-5-199909070-00003

[102] Van GB, Hultdin J, Johansson I, Witthoft C, Weinehall L, Eliasson M, et al Plasma folate and total homocysteine levels are associated with the risk of myocardial infarction, independently of each other and of renal function. J Intern Med. 2009 8;266(2):182–95. 10.1111/j.1365-2796.2009.02077.x 19298497

[103] Koh-Banerjee P, Franz M, Sampson L, Liu S, Jacobs DR Jr., Spiegelman D, et al Changes in whole-grain, bran, and cereal fiber consumption in relation to 8-y weight gain among men. Am J Clin Nutr. 2004 11;80(5):1237–45. 15531671 10.1093/ajcn/80.5.1237

[104] Liu S, Willett WC, Manson JE, Hu FB, Rosner B, Colditz G. Relation between changes in intakes of dietary fiber and grain products and changes in weight and development of obesity among middle-aged women. Am J Clin Nutr. 2003 11;78(5):920–7. 14594777 10.1093/ajcn/78.5.920

[105] Barton BA, Eldridge AL, Thompson D, Affenito SG, Striegel-Moore RH, Franko DL, et al The relationship of breakfast and cereal consumption to nutrient intake and body mass index: the National Heart, Lung, and Blood Institute Growth and Health Study. J Am Diet Assoc. 2005 9;105(9):1383–9. 10.1016/j.jada.2005.06.003 16129079

[106] Steffen LM, Jacobs DR Jr., Murtaugh MA, Moran A, Steinberger J, Hong CP, et al Whole grain intake is associated with lower body mass and greater insulin sensitivity among adolescents. Am J Epidemiol. 2003 8 1;158(3):243–50. 12882946 10.1093/aje/kwg146

[107] Pol K, Christensen R, Bartels EM, Raben A, Tetens I, Kristensen M. Whole grain and body weight changes in apparently healthy adults: a systematic review and meta-analysis of randomized controlled studies. Am J Clin Nutr. 2013 10;98(4):872–84. 10.3945/ajcn.113.064659 23945718

[108] Meyer KA, Kushi LH, Jacobs DR Jr., Slavin J, Sellers TA, Folsom AR. Carbohydrates, dietary fiber, and incident type 2 diabetes in older women. Am J Clin Nutr. 2000 4;71(4):921–30. 10731498 10.1093/ajcn/71.4.921

[109] Montonen J, Knekt P, Jarvinen R, Aromaa A, Reunanen A. Whole-grain and fiber intake and the incidence of type 2 diabetes. Am J Clin Nutr. 2003 3;77(3):622–9. 12600852 10.1093/ajcn/77.3.622

[110] Fung TT, Hu FB, Pereira MA, Liu S, Stampfer MJ, Colditz GA, et al Whole-grain intake and the risk of type 2 diabetes: a prospective study in men. Am J Clin Nutr. 2002 9;76(3):535–40. 12197996 10.1093/ajcn/76.3.535

[111] Barbagallo M, Dominguez LJ. Magnesium and type 2 diabetes. World J Diabetes. 2015 8 25;6(10):1152–7. 10.4239/wjd.v6.i10.1152 26322160 PMC4549665

[112] Gunther T. The biochemical function of Mg(2)+ in insulin secretion, insulin signal transduction and insulin resistance. Magnes Res. 2010 3;23(1):5–18. 10.1684/mrh.2009.0195 20228013

[113] Myers SA. Zinc transporters and zinc signaling: new insights into their role in type 2 diabetes. Int J Endocrinol. 2015;2015:167503 10.1155/2015/167503 25983752 PMC4423030

[114] Ludwig DS. The glycemic index: physiological mechanisms relating to obesity, diabetes, and cardiovascular disease. JAMA. 2002 5 8;287(18):2414–23. 11988062 10.1001/jama.287.18.2414

[115] Willett W, Manson J, Liu S. Glycemic index, glycemic load, and risk of type 2 diabetes. Am J Clin Nutr. 2002 7;76(1):274S–80S. 12081851 10.1093/ajcn/76/1.274S

[116] Tosh SM. Review of human studies investigating the post-prandial blood-glucose lowering ability of oat and barley food products. Eur J Clin Nutr. 2013 4;67(4):310–7. 10.1038/ejcn.2013.25 23422921

[117] Zammit VA, Waterman IJ, Topping D, McKay G. Insulin stimulation of hepatic triacylglycerol secretion and the etiology of insulin resistance. J Nutr. 2001 8;131(8):2074–7. 11481396 10.1093/jn/131.8.2074

[118] Joslowski G, Goletzke J, Cheng G, Gunther AL, Bao J, Brand-Miller JC, et al Prospective associations of dietary insulin demand, glycemic index, and glycemic load during puberty with body composition in young adulthood. Int J Obes (Lond). 2012 11;36(11):1463–71. 22249223 10.1038/ijo.2011.241

[119] Joslowski G, Halim J, Goletzke J, Gow M, Ho M, Louie JC, et al Dietary glycemic load, insulin load, and weight loss in obese, insulin resistant adolescents: RESIST study. Clin Nutr. 2015 2;34(1):89–94. 10.1016/j.clnu.2014.01.015 24534012

