# Supplementary material for: Effects of Ready-to-Eat-Cereals on Key Nutritional and Health Outcomes: A Systematic Review
Source: PLoS One. 2016 Oct 17;11(10):e0164931. doi: 10.1371/journal.pone.0164931 (PMC5066953; doi:10.1371/journal.pone.0164931)
Supplement: S2 Checklist — (DOCX) [file pone.0164931.s002.docx]

**S2 Checklist.** MOOSE Checklist

(from Stroup DF et al, Meta-analysis of observational studies in epidemiology: a proposal for reporting. Meta-analysis Of Observational Studies in Epidemiology (MOOSE) group. 2000 JAMA. 283: 2008-2012.)

| **Reporting of background should include** | Reported on page |
| --- | --- |
| Problem definition | 4 |
| Hypothesis statement | 4 |
| Description of study outcome(s) | 4-5 |
| Type of exposure or intervention used | 4-5 |
| Type of study designs used | 4-5 |
| Study population | 4-5 |
| **Reporting of search strategy should include** |  |
| Qualifications of searchers (eg, librarians and investigators) | 5 |
| Search strategy, including time period included in the synthesis and keywords | 5 |
| Effort to include all available studies, including contact with authors | 5 |
| Databases and registries searched | 5, S1 Protocol |
| Search software used, name and version, including special features used (eg, explosion) | No software used |
| Use of hand searching (eg, reference lists of obtained articles) | 5 |
| List of citations located and those excluded, including justification | Figure 1 |
| Method of addressing articles published in languages other than English | 7, S1 Protocol |
| Method of handling abstracts and unpublished studies | 5 |
| Description of any contact with authors | 5 |
| **Reporting of methods should include** |  |
| Description of relevance or appropriateness of studies assembled for assessing the hypothesis to be tested | 5-6 |
| Rationale for the selection and coding of data (eg, sound clinical principles or convenience) | 5-6 |
| Documentation of how data were classified and coded (eg, multiple raters, blinding, and interrater reliability) | 5-6 |
| Assessment of confounding (eg, comparability of cases and controls in studies where appropriate) | 6 |
| Assessment of study quality, including blinding of quality assessors; stratification or regression on possible predictors of study results | 6 |
| Assessment of heterogeneity | Not applicable (NA), as no meta-analysis |
| Description of statistical methods (eg, complete description of fixed or random effects models, justification of whether the chosen models account for predictors of study results, dose-response models, or cumulative meta-analysis) in sufficient detail to be replicated | NA, as no meta-analysis |
| Provision of appropriate tables and graphics | Table 1 - 3 |
| **Reporting of results should include** |  |
| Graphic summarizing individual study estimates and overall estimate | NA, as no meta-analysis |
| Table giving descriptive information for each study included | Table 1 - 3 |
| Results of sensitivity testing (eg, subgroup analysis) | NA, as no meta-analysis |
| Indication of statistical uncertainty of findings | NA, as no meta-analysis |
| **Reporting of discussion should include** |  |
| Quantitative assessment of bias (eg, publication bias) | Figure 2 |
| Justification for exclusion (eg, exclusion of non–English-language citations) | Figure 1 |
| Assessment of quality of included studies | 8, 50 |
| **Reporting of conclusion should include** |  |
| Consideration of alternative explanations for observed results | 44-49 |
| Generalization of the conclusions | 50 |
| Guidelines for future research | 50 |
| Disclosure of funding source | online |
