# Supplementary material for: Effects of Ready-to-Eat-Cereals on Key Nutritional and Health Outcomes: A Systematic Review
Source: PLoS One. 2016 Oct 17;11(10):e0164931. doi: 10.1371/journal.pone.0164931 (PMC5066953; doi:10.1371/journal.pone.0164931)
Supplement: S1 Protocol — (DOC) [file pone.0164931.s003.doc]

**Working title: Summary of the evidence on the effects of ready-to-eat-cereals on key nutritional and health benefits**

MG Priebe^1^, J McMonagle^2^

^1^Nutrition Reviewed, Murnau, Germany

^2^Cereal Partners Worldwide, Lausanne, Switzerland

- **Protocol, 07 November 2014 -**

**Content**

page

**A. BACKGROUND 3**

**B. OBJECTIVES 3**

**C. CRITERIA FOR CONSIDERING STUDIES FOR THIS REVIEW**

**Objective 1: Nutritional benefits 4**

**1. Types of studies**

**2. Types of participants**

**3. Types of interventions**

**4. Types of outcome measures**

**Objective 2: Health benefits 5**

**1. Types of studies**

**2. Types of participants**

**3. Types of interventions**

**4. Types of outcome measures**

**D. SEARCH STRATEGY FOR IDENTIFICATION OF STUDIES 6**

**E. METHODS OF THE REVIEW 7**

**1. Study selection**

**2. Quality assessment of studies**

**3. Data extraction**

**4. Data analysis**

**A. BACKGROUND**

Breakfast is a very important meal of the day. It has been shown that eating breakfast compared to skipping breakfast has beneficial effects on. In many countries breakfast cereals (BC) are considered the main component of a balanced breakfast. There are a considerable number of studies conducted to investigate the impact of the consumption of BC on nutritional and health benefits. However, the group of BC comprises many different cereal products and can be divided roughly into cooked cereals, like porridge type breakfasts, and ready-to-eat cereals (RTEC) or “cold” breakfast cereals like corn flakes and muesli. It is obvious that nutritional and health benefits depend on the composition of the breakfast meal. Many observational studies do not differentiate between RTEC and cooked cereals and in intervention trials BC are often either only part of breakfast or consumed not only for breakfast. To obtain more specifically information on nutritional and health benefits of cereals consumed at breakfast it is necessary to consider the specific composition of BC while summarizing and evaluating the available evidence. Therefore, in this systematic review, studies are included that investigate the effect of RTEC only and an attempt is made to relate their specific composition to specific health benefits.

**B. OBJECTIVES**

**1. To assess whether consumption of RTEC is associated with recommended energy intake, macronutrient intake, fiber intake and micronutrient intake/status using all available cohort studies and (randomized) controlled trials in children, adolescents and adults.**

**2. To systematically review and summarize the effects of consumption of RTEC on key health parameters in healthy persons or persons at risk using all available prospective cohort studies and (randomized) controlled trials.**

Key health parameters considered:

- Energy Management (i.e. glycemic response, food intake and satiety)

- Weight management (i.e. BMI, body weight, central and visceral adiposity, waist circumference)

- Cardiovascular Health (i.e. dyslipidemia, hypertension)

- Digestion/Gut Health (i.e. bowel function, coeliac disease, inflammatory bowel disease, gastro-intestinal pathogens)

- Immune function (i.e. higher vaccination responses, inflammatory response, infection)

- Performance (i.e. mental and physical)

- Bone growth and development (i.e. bone growth relating to kids and osteoporosis relating to the aging population)

Definition RTEC: RTEC comprise all cold breakfast cereals

**C. CRITERIA FOR CONSIDERING STUDIES FOR THIS REVIEW**

**Objective 1: Nutritional benefits**

**1. Types of studies**

Randomized controlled trials, controlled trials and cohort studies will be considered.

**2. Types of participants**

(Randomized) controlled trials:

***Inclusion criteria***

Healthy persons and persons with risk factors of disease (adults, adolescents, children)

Cohort studies:

***Inclusion criteria***

Healthy free living persons (adults, adolescents, children) and persons with risk factors of disease.

**3. Types of interventions**

(Randomized) controlled trials:

All trials (acute, medium and long-term) that investigate the effects of RTEC.

Cohort studies:

The intake of RTEC according to the definition is assessed.

**4. Types of outcome measures**

(Randomized) controlled trials:

- Energy intake

- Macronutrient intake

- Micronutrient intake/status

- Fiber intake

Cohort studies:

- Energy intake

- Macronutrient intake

- Micronutrient intake/status

- Fiber intake

**Objective 2: Health benefits**

**1. Types of studies**

Randomized controlled trials, controlled trials and prospective cohort studies will be considered.

**2. Types of participants**

(Randomized) controlled trials:

***Inclusion criteria***

Healthy persons and persons with risk factors of disease (adults, adolescents, children)

Prospective cohort studies:

***Inclusion criteria***

Healthy free living persons (adults, adolescents, children) and persons with risk factors of disease.

**3. Types of interventions**

(Randomized) controlled trials:

All trials (acute, medium- and long-term) that investigate the effects of RTEC:

either comparing different amounts of RTEC or different types of RTEC (e.g. high/low GI RTEC, high/low dietary fiber RTEC)

Prospective cohort studies:

The intake of RTEC according to the definition is assessed.

**4. Types of outcome measures**

(Randomized) controlled trials:

- Energy Management (i.e. glycemic response, satiety and food intake)

- Weight management (i.e. BMI, body weight, central and visceral adiposity, waist circumference)

- Cardiovascular Health (i.e. dyslipidemia, hypertension)

- Digestion/Gut Health (i.e. bowel function, coeliac disease, inflammatory bowel disease, gastro-intestinal pathogens)

- Immune function (i.e. higher vaccination responses, inflammatory response, infection)

- Performance (i.e. mental and physical),

- Bone growth and development (i.e. bone growth relating to kids and osteoporosis relating to the aging population)

Prospective cohort studies:

- Energy Management

- Weight management,

- Cardiovascular Health

- Digestion/Gut Health

- Immune function

- Performance

- Bone growth and development

**D. SEARCH STRATEGY FOR IDENTIFICATION OF STUDIES**

**1. Electronic searches**

The following data bases will be searched with no time limit and no language restriction:

- Cochrane Central Register of Controlled Trials (http://www.thecochranelibrary.com)

- MEDLINE (http://www.ncbi.nlm.nih.gov/pubmed)

- EMBASE ([https://www.embase.com](https://www.embase.com/))

- CINAHL (http://web.a.ebscohost.com.proxy-ub.rug.nl/ehost/search/advanced?sid=ea241342-c2cd-4e25-934e-f4ecd10a1d90%40sessionmgr4002&vid=0&hid=4109)

The following MEDLINE search strategy will be adapted for the other electronic databases searched. Search terms are free text terms.

Articles published in other languages than English will be translated into English by a suitable person.

**Dietary intervention:**

1. Breakfast OR fortified OR ready-to-eat

2. Cereal OR cereals

3. 1 AND 2

**Nutrition/health outcomes:**

No specific search item will be used for nutrition and health outcomes as we aim for a very wide range.

**2. Hand searches**

The reference lists of all included studies and of review articles will be searched in order to identify possible studies of interest.

**3. Additional searches**

Additional key words of relevance may be identified during any of the electronic or other searches. If this is the case, electronic search strategies will be modified to incorporate these terms.

**E. METHODS OF THE REVIEW**

**1. Study selection**

The title and abstract of each record of the search will be assessed by two reviewers (MP, JM) independently. Studies will be rejected if the article definitely does not meet the review's inclusion criteria, otherwise the full text of the study will be obtained. Differences between reviewers' results will be resolved by discussion and, when necessary, in consultation with a third party (CE).

**INCLUSION CRITERIA**

(Randomized) controlled trials

Population: Healthy persons and persons with risk factors of disease (adults, adolescents, children)

Intervention: consumption of RTEC alone and in combination with milk and fruit only

Definition: RTEC comprise all cold breakfast cereals

Outcome:

- Energy intake

- Macronutrient intake

- micronutrient intake/status

- fiber intake

Outcomes related to:

- Energy Management (i.e. glycemic response, satiety and food intake)

- Weight management (i.e. BMI, body weight, central and visceral adiposity, waist circumference),

- Cardiovascular Health (i.e. dyslipidemia, hypertension),

- Digestion/Gut Health (i.e. bowel function, gastro-intestinal pathogens),

- Immune function (i.e. higher vaccination responses, inflammatory response, infection)

- Performance (i.e. mental and physical),

- Bone growth and development (i.e. bone growth relating to kids and osteoporosis relating to the aging population).

Prospective cohort studies/ cross-sectional studies:

Population: health persons or person with risk factors of disease

Food intake assessed: consumption of RTEC

Outcome:

- Energy intake

- Macronutrient intake

- Micronutrient intake/status

- Fiber intake

Prospective cohort studies only:

Outcomes related to:

- Energy Management

- Weight management

- Cardiovascular Health

- Digestion/Gut Health

- Immune function

- Performance

- Bone growth and development

**EXCLUSION CRITERIA**

(Randomized) controlled trials:

- breakfast skippers vs breakfast eaters

- persons consuming hypocaloric diets

- intervention included cooked breakfast cereals

- breakfast included other products than RTEC, mik and fruit

- RTEC not (only) eaten at breakfast or if eaten as “meal replacer”

- if outcome is postprandial glucose or insulin response: breakfasts with different carbohydrate content

Prospective studies:

- breakfast skippers vs breakfast eaters

- “Breakfast cereals” are not defined

- definition of “breakfast cereals” includes cooked cereals

Cross-sectional studies:

- breakfast skippers vs breakfast eaters

- “Breakfast cereals” are not defined

- definition of “breakfast cereals” includes cooked cereals

**2. Assessment risk of bias**

Risk of bias will be assessed by one reviewer (MP).

(Randomized) controlled trials

According to The Cochrane Collaboration’s tool for assessing risk of bias (“low”, “high” or “unclear risk”):

Selection bias: random sequence generation, allocation concealment,

Performance bias: blinding participants and personnel,

Detection bias: blinding of outcome assessment,

Attrition bias: incomplete outcome data,

Reporting bias: selective reporting,

Other bias (only cross-over studies): appropriateness and methodology of cross-over design in

Cohort studies:

- methods for selecting study participants,

- number of appropriate confounders investigated and adjusted for;

- quality of method used to assess dietary intake, e.g. food frequency questionnaire with/without validation,

- quality of method used to assess outcome measures: e.g. self-report with/without validation, or direct measurement/medical records

- duration/completeness of follow-up (prospective studies only).

**3. Data extraction**

Data will be extracted from original reports by one reviewer (MP) using data extraction forms applied in the following Cochrane review: Priebe MG, van Binsbergen JJ, de VR, Vonk RJ. Whole grain foods for the prevention of type 2 diabetes mellitus. Cochrane Database Syst Rev. 2008;(1):CD006061.

The following items will be extracted:

(Randomized) controlled trials

1. General information: title, authors, country, year of publication, funding, duplicate publication;

2. Trial characteristics: design, duration, randomizations, concealment of allocation, blinding, checking of blinding;

3. Intervention: length of intervention, dietary advice/diet provided, comparison interventions;

4. Participants: population, exclusion criteria, number (total, per compared groups), age, gender, health condition; diagnostic criteria used to define health condition, similarity groups at baseline, assessment of compliance, withdrawals/losses to follow-up;

5. Outcomes: outcomes specified above (primary and secondary outcomes of the studies);

6. Results: for outcomes and times of assessment (including a measure of variation), intention-to-treat analyses.

Cohort studies:

1. General information: title, authors, country, year of publication, duplicate publication;

2. Study characteristics: design, dates of enrolment, follow-up;

3. Exposure: type, type of measurement, validation of measurement, time-points measurements;

4. Outcome: type, criteria used, type of measurement, validation of measurement;

5. Participants: number, characteristics;

6. Results: total number of cases, cases in group with lowest and highest intake, results of outcome, confounders adjusted for.

**4. Data analysis**

Data will not be summarized statistically as there are expected to be not sufficient similar.
