# Supplementary material for: Effects of Ready-to-Eat-Cereals on Key Nutritional and Health Outcomes: A Systematic Review
Source: PLoS One. 2016 Oct 17;11(10):e0164931. doi: 10.1371/journal.pone.0164931 (PMC5066953; doi:10.1371/journal.pone.0164931)
Supplement: S1 Table — (DOCX) [file pone.0164931.s004.docx]

**S1 Table. Differences in daily intake of energy, macronutrients, cholesterol, dietary fiber and sodium of frequent versus low/no RTEC consumers**

| Study | Population  age |  | Energy | Dietary fat  g (%en) | Saturated fat g (%en) | Cholesterol  mg | Carbo-  hydrates  g (%en) | Total sugars  g (%en) | DF/WG  g/g | Sodium  mg | Protein  g (%en) |
| --- | --- | --- | --- | --- | --- | --- | --- | --- | --- | --- | --- |
| **Children/adolescents** | | | | | | | | | | | |
| Albertson et al, 2003 [36] | 4 – 12 y |  | - | ↓ (NA) | - (NA) | ↓ | - (NA) | - (NA) | -/NA | - | - (NA) |
| Albertson et al, 2011 [27] | 6 – 18 y |  | ↑ | ↑ (NA) | - (NA) | ↓ | ↓ (NA) | ↑ (NA) | ↑/↑ | - | ↑ (NA) |
| Albertson et al, 2013 [44] | 4 – 12 y  Food secure |  | - | ↓ (NA) | NA (NA) | ↓ | ↑ (NA) | - (NA) | ↑/↑ | - | - (NA) |
| Albertson et al, 2013 [44] | 4 – 12 y  Not fully food secure |  | - | ↓ (NA) | NA (NA) | ↓ | ↑ (NA) | - (NA) | ↑/↑ | - | - (NA) |
| Balvin et al, 2013 [51] | 9.1 ± 0.5 y (mean±sd) |  | - | - (NA) | - (NA) | ↓ | - (NA) | NA (NA) | -/NA | - | NA (NA) |
| Barr et al, 2014 [22] | 4 – 18 y  men |  | - | ↓ (NA) | ↓ (NA) | ↓ | ↑ (NA) | ↑ (NA) | ↑/NA | NA | - (NA) |
| Barr et al, 2014 [22] | 4 – 18 y  women |  | - | ↓ (NA) | ↓ (NA) | ↓ | ↑ (NA) | ↑ (NA) | ↑/NA | NA | - (NA) |
| Deshmukh-Taskar et al 2010 [29] | 9 - 13 y |  | - | ↓ (NA) | - (NA) | ↓ | ↑ (NA) | ↑ (NA) | ↑/NA | ↓ | - (NA) |
| Deshmukh-Taskar et al 2010 [29] | 14 – 18 y |  | - | ↓ (NA) | ↓ (NA) | ↓ | ↑ (NA) | ↑ (NA) | ↑/NA | ↓ | - (NA) |
| Grieger et al, 2012 [26] | 12 – 16 y  boys |  | - | ↓ (↓) | - (-) | - | ↑ (↑) | ↑ (↑) | ↑/NA | - | - (-) |
| Grieger et al, 2013 [45] | 2 – 16 y |  | NA | NA (NA) | NA (NA) | NA | NA (NA) | NA (NA) | ↑/NA | NA | NA (NA) |
| Kafatos et al, 2005 [35] | 15 ± 0.4 y |  | - | - (-) | - (-) | - | - (-) | NA (NA) | ↑/NA | - | - (-) |
| McNulty et al, 1996 [39] | 12 y boys |  | ↑ | NA (↑) | NA (NA) | NA | NA (-) | - (NA) | ↑/NA | NA | NA (-) |
| McNulty et al, 1996 [39] | 12 y girls |  | - | NA (↑) | NA (NA) | NA | NA (↑) | ↑ (NA) | ↑/NA | NA | NA (-) |
| McNulty et al, 1996 [39] | 15 y boys |  | ↑ | NA (↑) | NA (NA) | NA | NA (-) | ↑ (NA) | ↑/NA | NA | NA (↑) |
| McNulty et al, 1996 [39] | 15 y girls |  | - | NA (-) | NA (NA) | NA | NA (-) | - (NA) | ↑/NA | NA | NA (-) |
| MontenegroBethancourt et al, 2009 [30] | 8 – 10 y  boys |  | ↑ | ↑ (NA) | NA (NA) | NA | - (NA) | NA (NA) | NA/NA | NA | ↑ (NA) |
| Nicklas et al, 1995 [42] | 10 y |  | - | - (-) | - (-) | - | - (-) | - (NA) | NA/NA | NA | - (-) |
| Ortega et al, 1996 [41] | 9 – 13 y |  | - | - (↓) | NA (-) | - | ↑ (↑) | NA (NA) | NA/NA | - | - (-) |
| Papoutsou et al, 2014 [20] | 4 – 8 y |  | - | - (↓) | - (NA) | ↓ | ↑ (↑) | NA (NA) | -/NA | - | - (NA) |
| Preziosi et al, 1999 [38] | 2 – 18 y |  | NA | NA (↓) | NA (NA) | NA | NA (↑) | NA (NA) | NA/NA | NA | NA (NA) |
| Ruxton et al, 1996 [40] | 7 – 8 y |  | - | - (↓) | NA (NA) | NA | ↑ (↑) | ↑ (↑) | ↑/NA | NA | - (-) |
| Williams et al, 2009 [31] | 1 – 12 y |  | - | ↓ (↓) | - (-) | ↓ | ↑ (↑) | ↑ (↑) | -/NA | - | ↓ (↓) |
| Koo et al, 2014 [21] | 10 – 11 y |  | ↑ | - (NA) | NA (NA) | NA | ↑ (NA) | NA (NA) | ↑/NA | NA | - (NA) |
| Morgan et al, 1986 [48] | 1 – 4 y |  | NA | - (NA) | NA (NA) | ↓ | NA (NA) | ↑ (NA) | NA/NA | ↑ | NA (NA) |
| Morgan et al, 1986 [48] | 5 - 12 y |  | NA | ↓ (NA) | NA (NA) | ↓ | NA (NA) | ↑ (NA) | NA/NA | ↓ | NA (NA) |
| Morgan et al, 1986 [48] | 13 – 17 y  men |  | NA | ↓ (NA) | NA (NA) | ↓ | NA (NA) | ↑ (NA) | NA/NA | - | NA (NA) |
| Morgan et al, 1986 [48] | 13 – 17 y  women |  | NA | - (NA) | NA (NA) | ↓ | NA (NA) | ↑ (NA) | NA/NA | - | NA (NA) |
| Morgan et al, 1981 [43] | 5 – 12 y |  | - | - (NA) | NA (NA) | ↓ | - (NA) | - (NA) | -/NA | - | - (NA) |
| Affenito et al, 2013 [23] | 5 - 18 y  SBP yes |  | - | NA (↓) | NA (↓) | ↓ | NA (NA) | - (NA) | ↑/↑ | - | - (NA) |
| Affenito et al, 2013 [23] | 5 - 18 y  SBP no |  | ↓ | NA (↓) | NA (-) | ↓ | NA (NA) | - (NA) | ↑/↑ | - | - (NA) |
| Albertson and Tobelmann, 1993 [7] | 7 – 12 y |  | - | ↓ (NA) | NA (NA) | ↓ | ↑ (NA) | ↑ (NA) | -/NA | - | - (NA) |
| Sampson et al, 1995 [47] | 7 – 10 y |  | NA | NA (↓) | NA (NA) | ↓ | NA (NA) | NA (NA) | NA/NA | - | NA (NA) |
| **Adults only or children/adolescents and adults** | | | | | | | | | | | |
| Van den Boom et al, 2006 [33] | 2 – 24 y |  | - | - (↓) | - (-) | - | ↑ (↑) | NA (NA) | -/NA | - | ↓ (↓) |
| Song et al, 2006 [32] | ≥ 4 y |  | - | NA (↓) | NA (NA) | NA | ↑ (↑) | NA (NA) | ↑/NA | NA | NA (-) |
| Albertson et al, 2013 [46] | ≥ 12 y |  | ↑ | - (NA) | - (NA) | - | ↑ (NA) | ↑ (NA) | ↑/NA | ↑ | ↑ (NA) |
| Preziosi et al, 1999 [38] | ≥ 18 y |  | NA | NA (↓) | NA (NA) | NA | NA (↑) | NA (NA) | NA (NA) | NA | NA (NA) |
| Barr et al, 2013 [24] | ≥ 19 y men |  | - | ↓ (NA) | ↓ (NA) | ↓ | ↑ (NA) | ↑ (NA) | ↑/NA | - | - (NA) |
| Barr et al, 2013 [24] | ≥ 19 y women |  | - | ↓ (NA) | ↓ (NA) | ↓ | ↑ (NA) | ↑ (NA) | ↑/NA | - | - (NA) |
| Song et al, 2005 [34] | ≥ 19 y  men |  | - | - (↓) | NA (NA) | NA | NA (↑) | NA (NA) | ↑/NA | NA | NA (NA) |
| Song et al, 2005 [34] | ≥ 19 y  women |  | - | - (↓) | NA (NA) | NA | NA (↑) | NA (NA) | ↑/NA | NA | NA (NA) |
| Nicklas et al, 1995 [42] | 19 – 28 y |  | - | - (↓) | - (-) | - | ↑ (↑) | ↑ (NA) | NA (NA) | NA | - (-) |
| Deshmukh-Taskar et al, 2010 [28] | 20 – 39 y |  | ↑ | NA (↓) | NA (↓) | ↓ | NA (↑) | NA (↑) | ↑/NA | ↑ | NA (↓) |
| Galvin et al, 2002 [37] | 18 – 64 y  men |  | NA | NA (↓) | NA (NA) | NA | NA (↑) | NA (↑) | ↑/NA | NA | NA (-) |
| Galvin et al, 2002 [37] | 18 – 64 y  women |  | NA | NA (↓) | NA (NA) | NA | NA (↑) | NA (↑) | ↑/NA | NA | NA (-) |
| Bertrais et al, 2000 [9] | 45 – 60 y  men |  | - | - (NA) | NA (NA) | NA | ↑ (NA) | ↑ (NA) | ↑/NA | NA | - (NA) |
| Bertrais et al, 2000 [9] | 35 – 60 y women |  | ↑ | - (NA) | NA(NA) | NA | ↑ (NA) | ↑ (NA) | ↑/NA | NA | ↑ (NA) |
| Albertson et al, 2012 [25] | ≥ 55 y |  | ↑ | ↓ (NA) | ↓ (NA) | ↓ | ↑ (NA) | ↑ (NA) | ↑/↑ | - | - (NA) |
| Wengreen et al, 2011 [53] | ≥ 65 y |  | ↑ | NA (↓) | ↓ (NA) | NA | NA (↑) | NA (NA) | ↑/↑ | NA | NA (↓) |

%en: percentage of total energy intake, - : no difference, DF: dietary fiber, NA: not assessed, SBP: School Breakfast Program, WG: whole grain
