# Supplementary material for: Effects of Ready-to-Eat-Cereals on Key Nutritional and Health Outcomes: A Systematic Review
Source: PLoS One. 2016 Oct 17;11(10):e0164931. doi: 10.1371/journal.pone.0164931 (PMC5066953; doi:10.1371/journal.pone.0164931)
Supplement: S2 Table — (DOCX) [file pone.0164931.s005.docx]

**S2 Table. Percentage of population with daily intake of micronutrients below recommended intake by frequency of RTEC consumption**

| **Study** | **Age group** | **Number of**  **partici-**  **pants^a^** | **Comparison^b^** | **Vitamin**  **A** | **VitaminC** | **Vitamin**  **E** | **Thiamin** | **Ribo-**  **flavin** | **Niacin** | **Vitamin**  **B 6** | **Folate** | **Vitamin B 12** | **Iron** | **Magnesium** | **Zinc** | **Calcium** | **Vitamin D** |
| --- | --- | --- | --- | --- | --- | --- | --- | --- | --- | --- | --- | --- | --- | --- | --- | --- | --- |
| **Percentages receiving less than the estimated average requirement (EAR)** | | | | | | | | | | | | | | | | | |
| **US** |  |  |  |  |  |  |  |  |  |  |  |  |  |  |  |  |  |
| Albertson et al, 2012 [25] | 55+ | 464  women | 0 vs  > 8 servings/14 d | **55/**  **18^#c^** | 55/  36^#^ | 97/  96 | 29/  5^#^ | 13/  0^#^ | 14/  2^#^ | **68/**  **13^#^** | **54/**  **4^#^** | 20/  1.0^#^ | 3/  0^#^ | 92/  68^#^ | **51/**  **14^#^** | 96/  77^#^ | 99/  99 |
| Albertson et al, 2012 [25] | 55+ | 431  men | 0 vs  > 8 servings/14 d | **58/**  **21^#^** | 58/  40^#^ | 95/  88^#^ | 15/  1^#^ | 4/  0^#^ | 4/  0^#^ | **41/**  **4^#^** | **26/**  **1^#^** | 7/  0^#^ | 2/  0 | 91/  70^#^ | **53/**  **16^#^** | **74/**  **34^#^** | 96/  87^#^ |
| Affenito et al, 2013 [23] | 5 – 18 y | 375 SBP  participants | No vs yes RTEC | 27.7/  11.1 | 9.9/  9.9 | NA | NA | NA | NA | NA | NA | NA | < 3/  < 3 | NA | NA | 59.0/  42.6 | NA |
| Affenito et al, 2013 [23] | 5 – 18 y | 1,571  SBP non- participants | No vs yes RTEC | 29.9/  10.3 | 14.7/  12.0 | NA | NA | NA | NA | NA | NA | NA | < 3/  < 3 | NA | NA | 67.1/  46.8 | NA |
| Albertson et al, 2011 [27] | 6 – 18 y | 8,848 | 0 vs  ≥ 1 serving/d | **42/**  **14** | 24/  15 | NA | 7/  0 | 4/  0 | 4/  0 | 10/  0 | **12/**  **0** | 3/  0 | 5/  0 | 46/  35 | **18/**  **3** | **71/**  **53** | NA |
| Albertson et al, 2003 [36] | 4 – 12 y | 603 | ≤3 vs ≥ 8 servings/14 d | **14/**  **0^#^** | 8/  2 | 55/  57 | 1/  0 | 1/  0 | 2/  0 | 3/  0 | **59/**  **9^#^** | NA | 1/  0 | **19/**  **9** | **10/**  **1^#^** | NA | NA |
| Song et al, 2006 [32] | 4 – 8 y  Men  9 – 13 y  14 – 18 y  Women  9 – 13 y  14 – 18 y | 772  539  680  562  634 | No vs yes RTEC | NA | NA | NA | NA | NA | NA | NA | NA | NA | NA | NA | NA | 63.1/38.4^#^  86.6/70.2^#^  66.9/53.2^#^  86.3/86.4^#^  86.7/69.1^#^ | NA |
| Song et al, 2006 [32] | Men  19 – 30 y  31 – 50 y  51 – 70 y  ≤ 71y  Women  19 – 30 y  31 – 50 y  51 – 70 y  ≤ 71y | 455  649  637  355  423  675  654  368 | No vs yes RTEC | NA | NA | NA | NA | NA | NA | NA | NA | NA | NA | NA | NA | 60.8/27.7^#^  65.1/37.9^#^  84.7/65.9^#^  82.8/73.3  77.8/49.0^#^  79.6/54.2^#^  91.6/77.4^#^  92.2/84.5 | NA |
| Albertson et al, 2013 [44] | 4 – 12 y | 1,755  Not fully food-secure | No vs yes RTEC | **33/**  **8** | 10/  5 | NA | 4/  0 | 4/  0 | 3/  0 | 6/  0 | 9/  0 | 2/  0 | 8/  0 | 26/  19 | **15/**  **3** | **63/**  **40** | **98/**  **87** |
| Albertson et al, 2013 [44] | 4 – 12 y | 2,982  Food secure | No vs yes RTEC | **27/**  **4** | 15/  7 | NA | 2/  0 | 2/  0 | 1/  0 | 5/  0 | 6/  0 | 1/  0 | 6/  0 | **26/**  **15** | **13/**  **1** | **60/**  **41** | **98/**  **87** |
| **Canada** |  |  |  |  |  |  |  |  |  |  |  |  |  |  |  |  |  |
| Barr et al, 2013 [24] | ≥19 y  men | 8,973 | No vs yes RTEC  Food only | 42/  38**^#^** | **27/**  **22** | NA | 2/  1 | 2/  1 | 0/  0 | 6/  4 | 5/  7 | 4/  2 | 0/  0 | **54/**  **33^#^** | 17/  13 | **53/**  **27^#^** | **92/**  **82^#^** |
| Barr et al, 2013 [24] | ≥19 y  women | 10,940 | No vs yes RTEC  Food only | 39/  33**^#^** | 15/  10**^#^** | NA | 8/  1 | 3/  1 | 0/  0 | **19/**  **11** | 20/  20 | 9/  10 | **20/**  **8** | **43/**  **26^#^** | 13/  9 | **72/**  **50^#^** | 98/  93 |
| Barr et al, 2014 [22] | 4 – 18 y  men | 5,526 | No vs yes RTEC  Food only | 19/  18 | 4/  5 | NA | 0/  0 | 0/  0 | 0/  0 | 1/  0 | 1/  2 | 1/  0 | 1/  0**^#^** | **20/**  **13** | 3/  3 | **43/**  **26^#^** | **81/**  **76** |
| Barr et al, 2014 [22] | 4 – 18 y  women | 5,281 | No vs yes RTEC  Food only | **24/**  **17** | 3/  1 | NA | 1/  0 | 1/  0 | 0/  0 | 5/  1 | 6/  3 | 3/  3 | 5/  1**^#^** | **32/**  **21^#^** | 11/  9 | **67/**  **43^#^** | 95/  89 |
| Albertson et al, 2013 [46] | 12 y and older | 2,926 | 0 - 1 vs  > 4 serving/wk | **58/**  **39^#^** | **55/**  **34^#^** | 97/  94^#^ | 13/  1^#^ | 9/  3^#^ | 9/  4^#^ | **39/**  **16^#^** | 26/  16^#^ | 15/  7^#^ | 13/  0^#^ | **78/**  **52^#^** | **42/**  **23^#^** | 71/  54^#^ | 96/  88^#^ |
| Ireland |  |  |  |  |  |  |  |  |  |  |  |  |  |  |  |  |  |
| Galvin et al, 2002 [37] | 18 - 64 y | 397  men | 0 vs on average 31 g/d | 24/  13 | 9/  2 | NA | NA | 21/  2 | NA | NA | 5/  0 | NA | 5/  0 | NA | 14/  5 | 15/  3 | NA |
| Galvin et al, 2002 [37] | 18 - 64 y | 419  women | 0 vs on average 27 g/d | 21/  16 | 15/  4 | NA | NA | 38/  0 | NA | NA | 19/  1 | NA | 55/  12 | NA | 22/  5 | 33/  10 | NA |
| **Percentages receiving less than two-thirds of the recommended dietary allowance (RDA)** | | | | | | | | | | | | | | | | | |
| USA |  |  |  |  |  |  |  |  |  |  |  |  |  |  |  |  |  |
| Nicklas et al, 1995 [42] | 10 y | 568 | No vs yes RTEC | 18/  3 | 34/  18 | 46/  50 | **31/**  **5** | 11/  2 | **28/**  **8** | **41/**  **10** | **19/**  **1** | 2/  3 | **35/**  **15** | 10/  8 | 27/  20 | 24/  16 | 50/  43 |
| Nicklas et al, 1995 [42] | Young  adults | 504 | No vs yes RTEC | **48/**  **16** | 52/  34 | 46/  45 | **51/**  **13** | 41/  9 | 20/  9 | **56/**  **18** | **53/**  **16** | 20/  9 | 49/  28 | 55/  34 | 51/  28 | **55/**  **12** | 69/  42 |
| Spain |  |  |  |  |  |  |  |  |  |  |  |  |  |  |  |  |  |
| Van den Boom et al, 2006  [33] | 2 – 24 y | 1,346  males | 0 vs > 40 g/d | **69/**  **64^#^** | **11/**  **3#** | **43/**  **38** | 0/  0# | 1/  1 | 0/  0 | 1/  0 | **12/**  **5#** | 0/  0 | 0/  0 | **6/**  **2** | NA | 2/  1# | 96/  97 |
| Van den Boom et al, 2006  [33] | 2 – 24 y | 1,506  females | 0 vs > 40 g/d | **66/**  **52#** | **10/**  **4#** | **71/**  **65#** | 0/  0 | 0/  0 | 0/  0 | 5/  2 | **71/**  **51#** | 0/  0 | **29/**  **12#** | 6/  3# | NA | **7/**  **1#** | 99/  98 |
| Cyprus |  |  |  |  |  |  |  |  |  |  |  |  |  |  |  |  |  |
| Papoutsou et al, 2014 [20] | 4 – 8 y | 250  RTEC vs  Other BF | No vs yes RTEC | 34/  15 | 27/  10 | NA | 6/  5 | NA | NA | 11/  7 | NA | NA | 37/  23 | 11/  10 | NA | 46/  34 | NA |
| **Percentages consuming less than 80 % of RDA** | | | | | | | | | | | | | | | | | |
| USA |  |  |  |  |  |  |  |  |  |  |  |  |  |  |  |  |  |
| Sampson et al, 1995 [47] | 7 – 10 y | 832  low-income AA | No vs yes RTEC | 64/  50# | 24/  15# | 49/  47 | 23/  6# | 14/  1# | **24/**  **4#** | **62/**  **28#** | **30/**  **2#** | 6/  2# | **45/**  **19#** | 27/  21 | NA | 42/  36 | 95/  85# |
| **Percentages consuming less than 100 % RDA** | | | | | | | | | | | | | | | | | |
| Albertson & Tobel-mann, 1993 [7] | 7 – 12 y | 487 | < 2 x vs > 7 times/14d | 50/  33 | 30/  11 | NA | **44/**  **9** | **35/**  **6** | **32/**  **7** | **92/**  **55** | NA | 3/  0 | 49/  17 | 57/  33 | 84/  77 | 71/  53 | NA |
| **Probability of not achieving 100 % of EAR** | | | | | | | | | | | | | | | | | |
| Australia |  |  |  |  |  |  |  |  |  |  |  |  |  |  |  |  |  |
| Grieger et al, 2012 [26] | 12 – 13 y | 184 | No vs yes RTEC | 1/  3 | NA | NA | 0/  0 | 1/  0 | 0/  0 | NA | **5/**  **0** | NA | 1/  0 | 0/  0 | 0/  0 | **68/**  **29^#^** | NA |
| Grieger et al, 2012 [26] | 14 – 16 y | 440 | No vs yes RTEC | **29/**  **8^#^** | NA | NA | 1/  0 | 1/  0 | 0/  0 | NA | **23/**  **1^#^** | NA | 2/  0 | 0/  0 | 15/  6 | **55/**  **18^#^** | NA |
| **Percentage who did not achieve LRNI** | | | | | | | | | | | | | | | | | |
| Ireland |  |  |  |  |  |  |  |  |  |  |  |  |  |  |  |  |  |
| McNulty et al, 1996 [39] | 12 y | 100 Boys | 0 vs > 40 g/d | NA | NA | NA | NA | 18/  0 | 18/  0 | NA | 100/  85 | 0/  0 | 0/  0 | NA | 0/  0 | 18/  0 | NA |
| McNulty et al, 1996 [39] | 15 y | 114 Boys | 0 vs > 40 g/d | NA | NA | NA | NA | 15/  0 | 20/  0 | NA | 75/  57 | 10/  0 | 0/  0 | NA | 0/  1 | 10/  0 | NA |
| McNulty et al, 1996 [39] | 12 y | 65 Girls | 0 vs > 40 g/d | NA | NA | NA | NA | 35/  0 | 13/  3 | NA | 94/  76 | 10/  0 | 35/  11 | NA | 23/  0 | 32/  3 | NA |
| McNulty et al, 1996 [39] | 15 y | 76 Girls | 0 vs > 40 g/d | NA | NA | NA | NA | 20/  0 | 8/  4 | NA | 96/  70 | 8/  0 | 20/  4 | NA | 2/  0 | 4/  0 | NA |

LRNI: lower reference nutrient intake, NA: not assessed, RDA: recommended dietary allowance

^a^ Number of participants from the results which are given in the table

^b^ Comparison lowest vs highest RTEC- intake

^c^ Bold: highest reduction of inadequacy

^#^significantly different
